# A Bayesian mixture model for clustering droplet-based single-cell transcriptomic data from population studies

**DOI:** 10.1038/s41467-019-09639-3

**Published:** 2019-04-09

**Authors:** Zhe Sun, Li Chen, Hongyi Xin, Yale Jiang, Qianhui Huang, Anthony R. Cillo, Tracy Tabib, Jay K. Kolls, Tullia C. Bruno, Robert Lafyatis, Dario A. A. Vignali, Kong Chen, Ying Ding, Ming Hu, Wei Chen

**Affiliations:** 1 Department of Biostatistics, Graduate School of Public Health, University of Pittsburgh, Pittsburgh, PA 15261 USA; 2 Department of Health Outcomes Research and Policy, Harrison School of Pharmacy, Auburn University, Auburn, AL 36849 USA; 3 Division of Pulmonary Medicine, Department of Pediatrics, Children’s Hospital of Pittsburgh of UPMC, University of Pittsburgh, Pittsburgh, PA 15224 USA; 4 School of Medicine, Tsinghua University, Beijing, 100084 China; 5 Department of Biostatistics, School of Public Health, University of Michigan, Ann Arbor, MI 48109 USA; 6 Department of Immunology, School of Medicine, University of Pittsburgh, Pittsburgh, PA 15262 USA; 7 Division of Rheumatology and Clinical Immunology, Department of Medicine, School of Medicine, University of Pittsburgh, Pittsburgh, PA 15261 USA; 8 School of Medicine, Tulane University, New Orleans, LA 70112 USA; 9 Tumor Microenvironment Center, UPMC Hillman Cancer Center, Pittsburgh, PA 15232 USA; 10 Cancer Immunology and Immunotherapy Program, UPMC Hillman Cancer Center, Pittsburgh, PA 15232 USA; 11 Division of Pulmonary, Allergy and Critical Care Medicine, Department of Medicine, School of Medicine, University of Pittsburgh, Pittsburgh, PA 15213 USA; 12 Department of Quantitative Health Sciences, Lerner Research Institute, Cleveland Clinic Foundation, Cleveland, OH 44195 USA

## Abstract

The recently developed droplet-based single-cell transcriptome sequencing (scRNA-seq) technology makes it feasible to perform a population-scale scRNA-seq study, in which the transcriptome is measured for tens of thousands of single cells from multiple individuals. Despite the advances of many clustering methods, there are few tailored methods for population-scale scRNA-seq studies. Here, we develop a Bayesian mixture model for single-cell sequencing (BAMM-SC) method to cluster scRNA-seq data from multiple individuals simultaneously. BAMM-SC takes raw count data as input and accounts for data heterogeneity and batch effect among multiple individuals in a unified Bayesian hierarchical model framework. Results from extensive simulation studies and applications of BAMM-SC to in-house experimental scRNA-seq datasets using blood, lung and skin cells from humans or mice demonstrate that BAMM-SC outperformed existing clustering methods with considerable improved clustering accuracy, particularly in the presence of heterogeneity among individuals.

## Abstract (web summary)

With the development of large scale single cell RNA-seq technology, population-scale scRNA-seq studies are emerging. Here, the authors develop BAMM-SC, a tool for clustering droplet-based scRNA-seq data from multiple individuals simultaneously.

## Introduction

Single-cell RNA sequencing (scRNA-seq) technologies have been widely used to measure gene expression for each individual cell, facilitating a deeper understanding of cell heterogeneity and better characterization of rare cell types^1,2^. Compared to early generation scRNA-seq technologies, the recently developed droplet-based technology, largely represented by the 10x Genomics Chromium system, has quickly gained popularity because of its high throughput (tens of thousands of single cells per run), high efficiency (a couple of days), and relatively lower cost (<$1 per cell)^3–6^. It is now feasible to conduct population-scale single-cell transcriptomic profiling studies, where several to tens or even hundreds of individuals are sequenced^7^.

A major task of analyzing droplet-based scRNA-seq data is to identify clusters of single cells with similar transcriptomic profiles. To achieve this goal, classic unsupervised clustering methods such as K-means clustering, hierarchical clustering, and density-based clustering approaches^8^ can be applied after some normalization steps. Recently, scRNA-seq tailored unsupervised methods, such as SIMLR^9^, CellTree^10^, SC3^11^, TSCAN^12^, and DIMM-SC^13^, have been designed and proposed for clustering scRNA-seq data. Supervised methods, such as MetaNeighbor, have been proposed to assess how well cell-type-specific transcriptional profiles replicate across different datasets^14^. However, none of these methods explicitly considers the heterogeneity among multiple individuals from population studies. In a typical analysis of population-scale scRNA-seq data, reads from each individual are processed separately and then merged together for the downstream analysis. For example, in the 10x Genomics Cell Ranger pipeline, to aggregate multiple libraries, reads from different libraries are downsampled such that all libraries have the same sequencing depth, leading to substantial information loss for individuals with higher sequencing depth. Alternatively, reads can be naively merged across all individuals without any library adjustment, leading to batch effects and unreliable clustering results.

Similar to the analysis of other omics data, several computational approaches have been proposed to correct batch effects for scRNA-seq data. For example, Spitzer et al.^15^ adapted the concept of force-directed graph to visualize complex cellular samples via Scaffold (single-cell analysis by fixed force- and landmark-directed) maps, which can overlay data from multiple samples onto a reference sample(s). Recently, two new methods: mutual nearest neighbors^16^ (MNN) (implemented in scran) and canonical correlation analysis (CCA)^17^ (implemented in Seurat) were published for batch correction of scRNA-seq data. All these methods require the raw counts to be transformed to continuous values under different assumptions, which may alter the data structure in some cell types and lead to difficulty of biological interpretation.

**Fig. 1:**
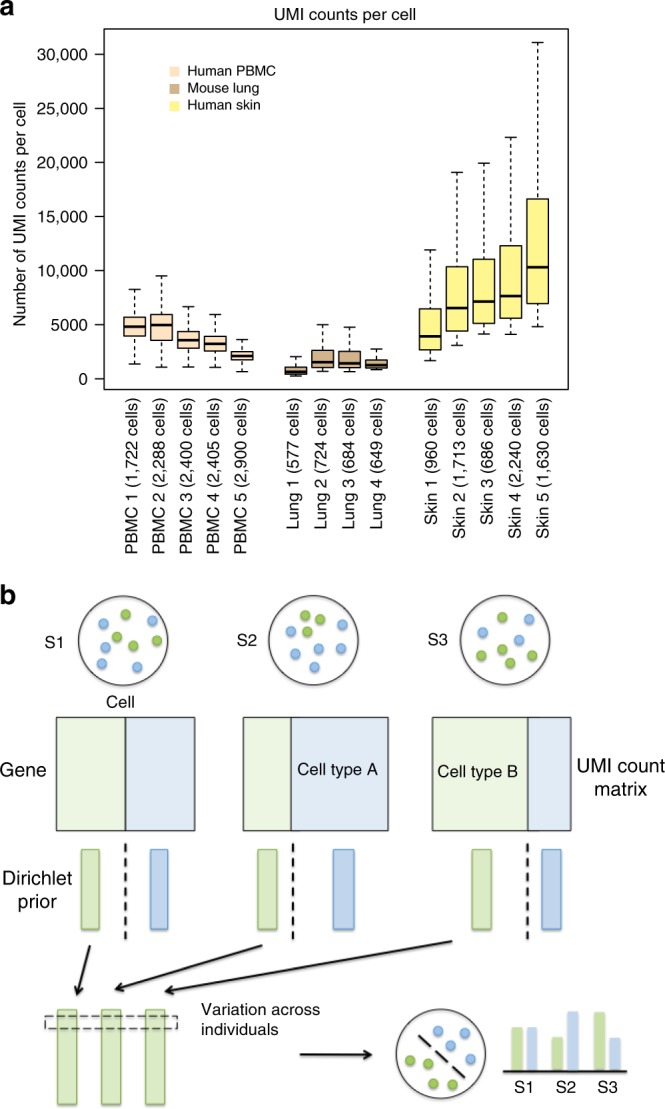
Sample information of real scRNA-seq datasets and the model structure in BAMM-SC. **a** UMI counts per cell of three droplet-based scRNA-seq datasets. In the boxplots, the box spans from the first to third quartile (depicting median as a line in the middle), the whiskers extend to 1.5× IQR (interquartile range). **b** An overall workflow of BAMM-SC

We first conducted an exploratory data analysis to demonstrate the existence of batch effect in multiple individuals using both publicly available and three in-house synthetic droplet-based scRNA-seq datasets, including human peripheral blood mononuclear cells (PBMC), mouse lung and human skin tissues. Detailed sample information was summarized in Fig. 1a and Supplementary Table 1. We use human PBMC as an example. We isolated from whole blood obtained from 4 healthy donors and used the 10x Chromium system to generate scRNA-seq data. We also included one additional healthy donor from a published PBMC scRNA-seq data^4^ to mimic the scenario where we combine the local dataset with the public datasets. In this cohort, sample 1 and sample 2 were sequenced in one batch; sample 3 and sample 4 were sequenced in another batch; sample 5 was downloaded from the original study conducted by 10x Genomics^4^. As an exploratory analysis, we produced a t-SNE plot based on the first 50 principal components (Supplementary Fig. 1) of all cells from these 5 donors and observed a clear batch effect: samples from the same batch tend to cluster together.

This illustrative example demonstrates the importance and urgent need for well characterizing different sources of variability and correcting potential batch effects among droplet-based scRNA-seq datasets collected from multiple individuals. In addition, due to the computational burden, many methods cannot be scaled up to analyze population-scale droplet-based scRNA-seq data with tens of thousands of cells collected from many individuals under various conditions. In this study, we propose a BAyesian Mixture Model for Single Cell sequencing (BAMM-SC) to simultaneously cluster large-scale droplet-based scRNA-seq data from multiple individuals. BAMM-SC directly works on the raw counts without any data transformation and models the heterogeneity from multiple sources by learning the distributions of signature genes in a Bayesian hierarchical model framework. In the following sections, we will describe our method, benchmark its performance against existing clustering methods in simulation studies, and evaluate our method for its accuracy, stability, and efficiency in three in-house synthetic scRNA-seq datasets including PBMCs, skin, and lung tissues from humans or mice.

## Results

### Overview of BAMM-SC

BAMM-SC represents a Bayesian hierarchical Dirichlet multinomial mixture model, which explicitly characterizes three sources of heterogeneity (i.e., genes, cell types, and individuals) (see Methods). Figure 1b provides an overview of the model structure in BAMM-SC, which directly models cell-type specific genes’ unique molecular identifier (UMI) counts and their heterogeneity among different individuals through a hierarchical distribution structure in a Bayesian framework. Our method has the following three key realistic assumptions. First, cell type clusters are discrete, and each cell belongs to one specific type exclusively. Second, heterogeneity exists among different individuals and across different cell types. The heterogeneity of the same cell type among different individuals is smaller than the heterogeneity across different cell types within the same individual. Third, cells of the same cell type share a similar gene expression pattern. That is, the underlying statistical distributions for cells within the same cell type are assumed to be the same. The mathematical model representations are included and explained in Supplementary Methods. Compared to other clustering methods which ignore individual level variability, BAMM-SC has the following four key advantages: (1) BAMM-SC accounts for data heterogeneity among multiple individuals, such as unbalanced sequencing depths and technical biases in library preparation, and thus reduces the false positives of detecting individual-specific cell types. (2) BAMM-SC borrows information across different individuals, leading to improved power for detecting individual-shared cell types and higher reproducibility as well as stability of the clustering results. (3) BAMM-SC performs one-step clustering on raw UMI count matrix without any prior batch-correction step, which is required for most clustering methods in the presence of batch effect. (4) BAMM-SC provides a statistical framework to quantify the clustering uncertainty for each cell in the form of posterior probability for each cell type (see Methods).

### Simulation studies

**Fig. 2:**
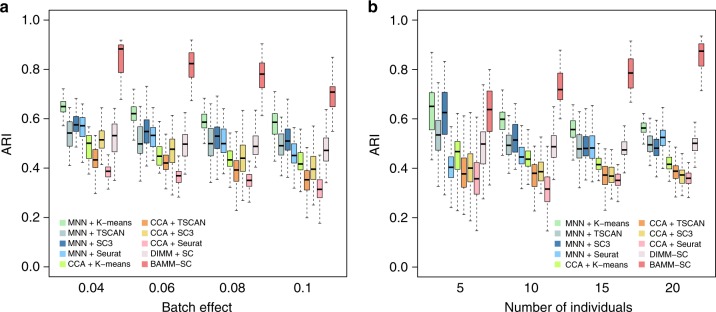
Boxplots of ARIs for 10 clustering methods across 100 simulations. **a** Investigating how different heterogeneities among multiple individuals (measured by mean $\sigma _{ik}^2$ values) affect clustering results. The simulated dataset consists of 10 individuals with 400 cells for each. **b** Investigating how different numbers of individuals affect clustering results. We set the level of heterogeneity (mean of $\sigma _{ik}^2$) among individuals as 0.1. In the boxplots, the box spans from the first to third quartile (depicting median as a line in the middle), the whiskers extend to 1.5× IQR (interquartile range)

We have conducted comprehensive simulation studies to benchmark the performance of BAMM-SC. Specifically, we simulated droplet-based scRNA-seq data collected from multiple individuals from the posited Bayesian hierarchical Dirichlet multinomial mixture model (see Methods and Supplementary Methods). We considered different experimental designs, including different heterogeneities among multiple individuals and different numbers of individuals (Fig. 2). In our posited hierarchical model, the log normal prior distribution LN $(\mu _{ik},\sigma _{ik}^2)$ measures the heterogeneity of gene *i* in cell type *k* among multiple individuals, where $\mu _{ik}$ and $\sigma _{ik}^2$ are related to the mean and variation of gene expression. Without loss of generality, we used the mean of $\sigma _{ik}^2$ across all genes and all cell types to quantify the overall individual level heterogeneity. We applied BAMM-SC to each synthetic dataset, and compared the inferred cell type label of each single cell with the ground truth, measured by adjusted Rand index (ARI)^18^. We compared BAMM-SC with other competing clustering methods (K-means, TSCAN, SC3, and Seurat), which are either methods from different clustering categories or recommended by recent reviews on clustering methods for single-cell data^19,20^. Since none of methods model batch effects and therefore each needs to be combined with a batch correction method as a preprocessing step in data analysis. We applied two recently published and prevalent methods srcan MNN^16^ and Seurat CCA^17^ prior to these clustering methods so that each combination can be a fair comparison with BAMM-SC, which does not need a separate batch correction step.

Specifically, we compared BAMM-SC with the other nine competing methods (MNN+K-means, MNN+TSCAN, MNN+SC3, MNN+Seurat, CCA+K-means, CCA+TSCAN, CCA+SC3, CCA+Seurat, and DIMM-SC) in the simulation studies. Noticeably, DIMM-SC, our previously developed method for clustering scRNA-seq data from a single individual, also takes the raw UMI count matrix as the input without any batch effect correction or data transformation. We pooled single cells from different individuals together while ignoring each individual label, and then applied DIMM-SC to the pooled data. We simulated 100 datasets and summarized the corresponding ARIs for each method.

As shown in Fig. 2a, BAMM-SC consistently outperformed the other nine competing methods across a variety of individual level heterogeneities by achieving higher average ARI and lower variation of ARI among 100 simulations. As expected, the performance of all ten clustering approaches decreases as the among individual heterogeneity increases, measured by the mean $\sigma _{ik}^2$ values. In Fig. 2b, with the increase of number of individuals, BAMM-SC achieved higher ARI, while ARIs of other methods either remained stable or decreased.

Furthermore, we performed comprehensive simulation studies by generating simulated scRNA-seq datasets from different number of cell type clusters (Supplementary Fig. 2a), different overall sequencing depths (Supplementary Fig. 2b), and different cell-type-specific heterogeneities (i.e., the mean difference of gene expression profiles between two distinct cell types) (Supplementary Fig. 2c). BAMM-SC consistently outperformed other methods in terms of accuracy and robustness in all these scenarios. Taken together, our comprehensive simulation studies have demonstrated that, when data are generated from the true model, BAMM-SC is able to appropriately borrow information across multiple individuals, account for unbalanced sequencing depths, and provide more accurate and robust clustering results than other competing methods.

**Fig. 3:**
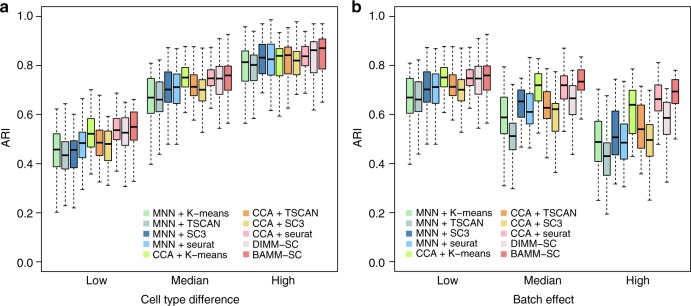
Boxplots of ARI for 10 clustering methods across 100 simulations using Splatter. **a** Investigating how different levels of group effect affect clustering results. We set the mean parameters of three cell types as (0.20, 0.21, 0.22), (0.20, 0.22, 0.24), and (0.20, 0.24, 0.28) to represent three levels (low, medium, and high) of group difference. **b** Investigating how different levels of batch effect affect clustering results. We set the mean parameters of the five individuals as (0.1, 0.1, 0.1, 0.1, 0.1), (0.12, 0.12, 0.12, 0.12, 0.12), and (0.14, 0.14, 0.14, 0.14, 0.14) to represent three levels (low, medium, and high) of batch effects. In the boxplots, the box spans from the first to third quartile (depicting median as a line in the middle), the whiskers extend to 1.5× IQR (interquartile range)

To evaluate the robustness of BAMM-SC when data generation model is mis-specified, we simulated additional datasets using R package Splatter^21^, a commonly used tool for scRNA-seq data simulation using a completely different model. To make our simulated data a good approximation to the real data, we first downloaded the raw UMI count matrix of a purified B-cell scRNA-seq dataset from the 10x Genomics website (https://support.10xgenomics.com/single-cell-gene-expression/datasets/1.1.0/b_cells), and used the function splatEstimate to estimate the parameters related to mean of gene, library size, expression outlier, dispersion across genes, and dropout rate. We assumed cell types are shared across multiple individuals, where each individual is treated as one batch with the same number of cells and genes. We further specified batch parameters and differential expression parameters to generate scenarios with different amount of group effect (i.e., cell type differences) and batch effect. As shown in Fig. 3, BAMM-SC still outperformed most other competing methods in terms of clustering accuracy in all scenarios, although the improvement is less substantial than our own model simulations, which is expected.

### Real data analysis on human PMBC dataset

For aforementioned human PBMC samples, we first pooled cells from five donors together, filtered lowly expressed genes that were expressed in less than 1% cells. We then extracted the top 1000 highly variable genes based on their standard deviations. As shown in Supplementary Fig. 3, we identified seven types of PBMCs based on the biological knowledge of cell-type-specific gene markers (Supplementary Table 2). Using these gene markers, >70% single cells can be assigned to a specific cell type. Since there is no gold standard for clustering analysis in this real dataset, we used the labels of these cells as the approximated ground truth to benchmark the clustering performance for different clustering methods. Cells with uncertain cell types were removed when calculating ARIs.

Similar to the simulation studies, we applied ten clustering methods on these samples and repeated each method ten times to evaluate the stability of its performance (Table 1). The total number of clusters was set as seven based on the biological knowledge from cell-type-specific gene markers. As shown in Table 1, BAMM-SC achieved the highest ARI for human PBMC samples compared to all other competing methods. Both TSCAN and Seurat are deterministic clustering methods and therefore they generate identical results for ten analyses.

**Table 1:** Performance of clustering across ten times analyses for three real datasets

Method	Mean_P	SD_P	Range_P	Mean_L	SD_L	Range_L	Mean_S	SD_S	Range_S
MNN+K-means	0.379	0.083	(0.283–0.485)	0.662	0.066	(0.596–0.815)	0.597	0.075	(0.461–0.676)
MNN+TSCAN	0.373	NA	NA	0.720	NA	NA	0.553	NA	NA
MNN+SC3	0.348	0.084	(0.266–0.511)	0.640	0.061	(0.556–0.687)	0.517	0.034	(0.436–0.557)
MNN+Seurat	0.325	NA	NA	0.749	NA	NA	0.647	NA	NA
CCA+K-means	0.414	0.056	(0.307–0.464)	0.695	0.114	(0.505–0.883)	0.619	0.129	(0.424–0.737)
CCA+TSCAN	0.210	NA	NA	0.611	NA	NA	0.398	NA	NA
CCA+SC3	0.145	0.052	(0.051–0.215)	0.610	0.068	(0.531–0.708)	0.369	0.071	(0.277–0.488)
CCA+Seurat	0.468	NA	NA	0.729	NA	NA	0.702	NA	NA
DIMM-SC	0.333	0.071	(0.302–0.529)	0.809	0.030	(0.742–0.868)	0.715	0.045	(0.671–0.779)
BAMM-SC	0.487	0.056	(0.362–0.532)	0.882	0.042	(0.764–0.910)	0.762	0.032	(0.717–0.843)

Columns Mean_P, SD_P, and Range_P were calculated from human PBMC dataset. Columns Mean_L, SD_L, and Range_L were calculated from mouse lung dataset. Columns Mean_S, SD_S, and Range_S were calculated from human skin dataset.

**Fig. 4:**
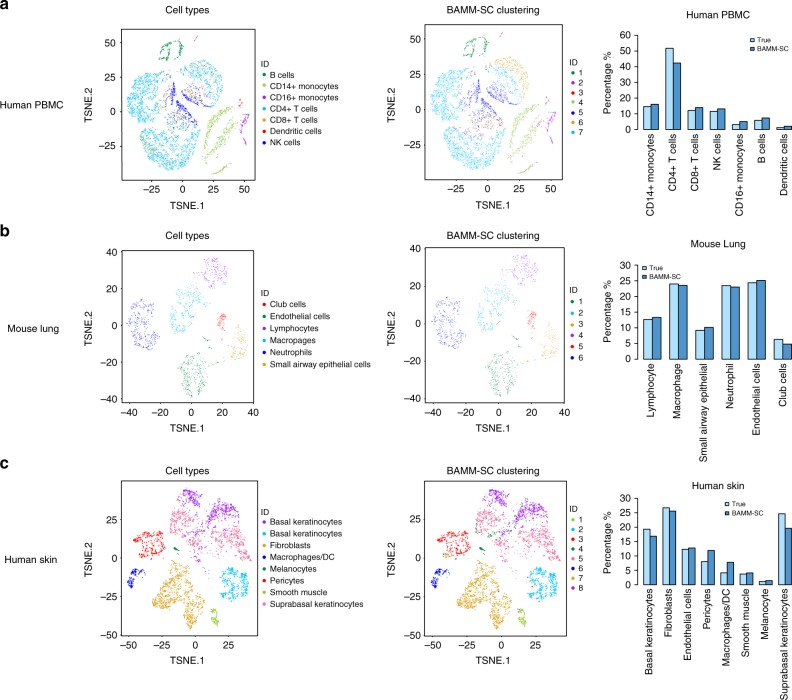
The performance of BAMM-SC clustering for three in-house scRNA-seq datasets. The t-SNE projection of cells (colored by the approximated truth and BAMM-SC clustering results) and bar plots of proportions of cell types among all individuals for **a** human PBMC, **b** mouse lung, and **c** human skin tissues, separately. BAMM-SC clustering labels are from the result with the highest ARI among ten times analysis

We further generated t-SNE plots with each cell colored by their cell-type classification based on specific gene markers (i.e., the approximated truth) (Fig. 4a (left figure)) and cluster labels inferred by BAMM-SC (Fig. 4a (middle figure)), respectively. Despite some dendritic cells were wrongly identified as CD16+Monocytes, these two plots are similar to each other (ARI = 0.532), suggesting that BAMM-SC performed well in human PBMC samples compared with other clustering methods.

Moreover, we calculated the averaged cell proportions of each cell type inferred from BAMM-SC among ten runs for five PBMC samples, compared with cell proportions calculated from the approximated truth based on gene markers. Figure 4a (right figure) shows that the proportions inferred from BAMM-SC are close to the truth, suggesting that BAMM-SC can adequately account for batch effect when clustering cells from multiple individuals. We also generated t-SNE projection plots colored by cluster labels inferred by other methods: MNN+K-means clustering (Supplementary Fig. 4a), MNN+TSCAN (Supplementary Fig. 4b), MNN+SC3 (Supplementary Fig. 4c), MNN+Seurat (Supplementary Fig. 4d), CCA+K-means (Supplementary Fig. 4e), CCA+TSCAN (Supplementary Fig. 4f), CCA+SC3 (Supplementary Fig. 4g), CCA+Seurat (Supplementary Fig. 4h), and DIMM-SC (Supplementary Fig. 4i).

### Real data analysis on mouse lung dataset

We collected lung mononuclear cells from four mouse samples under two conditions: *Streptococcus pneumonia* (SP) infected (sample 1 and 2) and naive (sample 3 and 4). Supplementary Figure 5 shows the t-SNE plot of lung mononuclear cells from four mouse samples. Similar to the analysis of PBMC samples, after filtering lowly expressed genes, we pooled cells from 4 mice together and extracted the top 1000 highly variable genes. As shown in Supplementary Fig. 6, we identified six types of cells based on the biological knowledge of cell-type specific gene markers (Supplementary Table 3). Taken together, >66% of single cells can be assigned to a specific cell type. Therefore, we used the labels of these cells as the approximated truth and removed cells with uncertain cell types from the downstream analysis.

Figure 4b (left figure) and Fig. 4b (middle figure) show the t-SNE plots with each cell colored by their cluster label based on cell-type-specific gene markers and cluster labels inferred by BAMM-SC, respectively. These two are highly similar (ARI = 0.910), indicating the outstanding performance of BAMM-SC. Table 1 shows that BAMM-SC considerably outperformed other nine clustering methods in terms of ARI. We also generated t-SNE plots colored by cluster labels inferred by other competing clustering methods (Supplementary Fig. 7). As shown in Supplementary Fig. 8, the proportions of neutrophils in SP infected samples (sample 1 and sample 2) are much higher than the proportions in naïve samples (sample 3 and sample 4). This is consistent with the fact that infections by bacteria and viruses may increase the number of neutrophils, which is a necessary reaction by the body^22,23^. Interestingly, the proportion of cell types in naïve sample 3 is different from others, which may due to unsatisfactory sample quality or unexpected bacterial infections.

### Real data analysis on human skin dataset

To evaluate the clustering performance of BAMM-SC in solid human tissues, we collected skin samples from five healthy donors that are part of a systemic sclerosis study^24^. Figure 1a and Supplementary Table 1 list the detailed sample information and Supplementary Fig. 9 shows the t-SNE plot of cells from five human skin samples after the data processing similar to previous analyses. As shown in Supplementary Fig. 10, we identified eight major types of cells based on the biological knowledge of cell-type-specific gene markers (Supplementary Table 4). Taken together, >67% of single cells can be assigned to a specific cell type. Similar to the other two real data analyses, we used the labels of these cells as the approximated truth and removed cells with uncertain cell types from the downstream analysis.

As shown in Fig. 3c, BAMM-SC performed well in human skin samples, since the t-SNE plot with each cell colored by their cell-type label based on gene markers is highly similar to the plot generated from the clustering result of BAMM-SC (ARI = 0.843). Also, BAMM-SC achieved higher ARI compared with all the other clustering methods (Table 1). As comparisons, we generated t-SNE plots colored by cluster labels inferred by different clustering (Supplementary Fig. 11).

### Other evaluation criteria

To further demonstrate the validity of BAMM-SC, we calculated the confusion matrix for three real datasets and reported the clustering accuracy (defined as the proportion of cells being classified into the correct cell-type cluster) (Supplementary Table 5, Supplementary Methods). Our method outperformed other competing methods in all three datasets. In addition, we performed a flow cytometry experiment, a gold standard method for quantifying cell population through cell surface markers, on the sample 3 from the human PBMC dataset, which has an additional aliquot from the same pool of cells. We used FlowJo software to gate each cell population through specific antibodies and calculated the percentage of each cell type. Then, we compared the proportions of different cell types from flow cytometry and the clustering result of BAMM-SC from scRNA-seq. Supplementary Figure 12 shows that the proportion of cells in each cell type classified by BAMM-SC is consistent with that being estimated by flow cytometry. We also calculated the Pearson’s correlation coefficient of cell proportions for each clustering method (Supplementary Table 6). Despite the different technology, the high correlation (Pearson correlation coefficient is 0.98) suggests that BAMM-SC is able to adequately account for heterogeneity among multiple individuals and provide reliable clustering results. To be noted, unlike other clustering methods we considered, Seurat cannot directly pre-specify the number of clusters K. Rather it needs to set a resolution parameter that indirectly controls the cluster number. In all three real data sets, after an extensive grid search, we found the resolution parameter that yields the same number of clusters as the one based on the biological knowledge. Therefore, for the two Seurat clustering methods, instead of using the clustering assignments that produced the highest ARI among ten times analysis, we computed the confusion matrix and the proportions of different cell types based on this specific resolution parameter.

It is challenging to evaluate clustering algorithms in experimental data since the ground truth of cell type label is generally unknown. Other than using ARI based on cell-type-specific gene markers as approximated ground truth, we also used cluster stability and tightness to evaluate the clustering performance. Specifically, we calculated the average proportion of nonoverlap (APN)^25^ clustered cells and silhouette width^26^ in three real datasets, respectively. APN is a cluster stability measurement which evaluates the stability of a clustering result by comparing it with the clusters obtained by removing one feature (i.e., one gene in our study) at a time. It measures the average proportion of observations not placed in the same cluster under both cases. To make computation affordable in our real data analysis, after extracting the top 1000 highly variable genes, we compared the clustering results based on the full data (1000 genes) to the clustering results based on a subset of data with 100 genes randomly removed. We repeated this step ten times to calculate the APN. For cluster tightness, the silhouette width ranges from −1 to 1, where a higher value indicates that the observation is better matched to its own cluster and worse matched to other clusters. For both measurements, BAMM-SC achieved high cluster stability and high cluster tightness in most scenarios, compared with all other competing methods (Supplementary Table 7, Supplementary Table 8).

### Uncertainty assessment

Different from other deterministic methods, BAMM-SC has the ability to assess clustering uncertainty through the posterior probability for each cell to belong to each cell-type cluster. As shown in Supplementary Fig. 13, we highlighted vague cells in the t-SNE projection plot, where vague cells are defined as cells with the largest posterior cluster-specific probability <0.95. In the human PBMC samples, most of the vague cells (colored in red) are located at the boundary of different clusters, which reassuring the validity of the clustering results. In real data analysis, users can decide to remove vague cells under a user-specified criterion (based on the posterior probability) for the downstream analysis such as differential gene expression analysis within each cell type.

## Discussion

In summary, we have developed a novel Bayesian framework for clustering population-scale scRNA-seq data. BAMM-SC retains the raw data information by directly modeling UMI counts without data transformation or normalization, facilitating straightforward biological interpretation. The Bayesian hierarchical model enables the joint characterization of multiple sources of uncertainty, including single-cell level heterogeneity and individual level heterogeneity. Furthermore, BAMM-SC can borrow information across different individuals through its mixture hierarchical model structure and Bayesian computational techniques, leading to improved clustering accuracy. BAMM-SC is based on probabilistic models, thus providing the quantification of clustering uncertainty for each single cell.

**Fig. 5:**
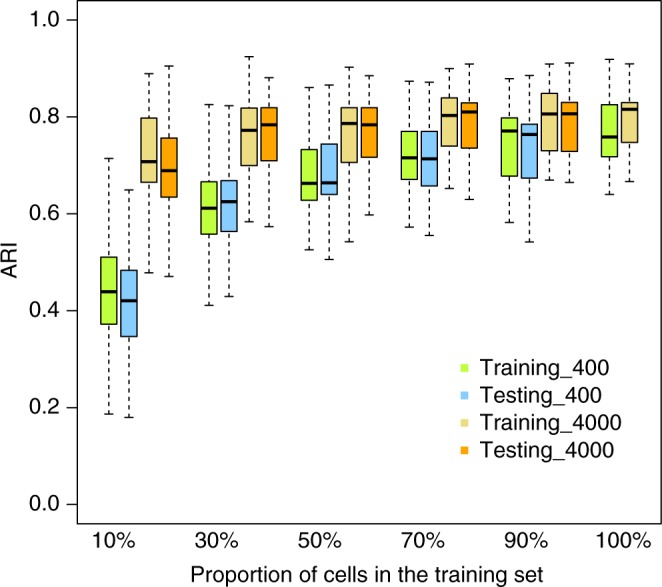
The Boxplots of ARI for BAMM-SC across 100 simulation. It demonstrates the clustering accuracy under different proportions of cells being selected in the training set. In the boxplots, the box spans from the first to third quartile (depicting median as a line in the middle), the whiskers extend to 1.5× IQR (interquartile range)

Our model coupled with a computationally efficient MCMC algorithm is able to cluster large-scale droplet-based scRNA-seq data with feasible computational cost. For example, using 1000 highly variable genes, it takes about 1.5, 2.5, and 4.5 h when analyzing the 3 real datasets (human PBMC, mouse lung and human skin), respectively. For the simulated dataset consist of 10 individuals with 4000 cells each, the computational time for clustering is about 30 min. Supplementary Figure 14 demonstrates that the computational time of BAMM-SC increases approximately linearly with the increase of the number of cells in each individual, the number of individuals and the number of clusters, respectively. To further improve the computational efficiency, we provided a supervised clustering option in BAMM-SC for very large-scale datasets. Specifically, users can first apply BAMM-SC on a small subset of single cells in each individual, and save predicted cluster labels as well as other informative parameters such as ${\boldsymbol{\alpha }}_{ \cdot {\boldsymbol{lk}}}$. Then for the remaining single cells, users can perform supervised classification via BAMM-SC instead of unsupervised clustering (see Methods). By clustering a small number of single cells, this procedure will substantially reduce the computational cost. We used the simulated dataset of ten individuals to demonstrate the effectiveness of this supervised option in Fig. 5. We simulated two datasets (Supplementary Methods): one dataset consists of 10 individuals with 400 cells each and the other dataset consists of 10 individuals with 4000 cells each. We selected a subset of cells in each individual as the training set and treated the remaining cells as the test set. We set the proportion of cells in the training set from 10 to 100% and reported the ARIs for both training and test sets. When the proportion equals 100%, there is no test data set, thus only ARI for the training set is reported. We repeated this simulation procedure 100 times and reported ARIs in Fig. 5 below. When the total number of cells in the training set is large enough (4000 in total or more), the prediction performance (measured by ARI) in the test set is saturated. For the dataset consists of 10 individuals with 4000 cells each, when we used 10% cells for training, it only takes ~90 s to obtain the clustering labels for all cells in both training and test sets with the similar performance from the full dataset. Therefore, for large datasets (e.g., >100 K cells), users can apply BAMM-SC to a smaller subset of cells in each individual to cluster distinct cell types, and then classify the remaining cells according to the predicted cell types. BAMM-SC is currently implemented in R/Rcpp with satisfactory computing efficiency to accommodate population scale scRNA-seq data. Further speed-up can be made through parallel computing or graphics processing unit.

In addition, we can predefine the number of clusters based on prior knowledge on the tissue or determine it using some standard model checking criterion such as Akaike's Information Criteria (AIC) or Bayesian Information Criteria (BIC). As shown in Supplementary Fig. 15, AIC and BIC work as expected in the analysis of simulated datasets and provide a reliable range of cluster numbers to guide real data analysis based on prior knowledge. However, in a biological study, the number of clusters is often considered as a continuum because of the nature of cell growth, so we recommend trying a range of cluster numbers in practice. BAMM-SC is shown to be robust against model mis-specification. In our simulation studies, we applied Splatter to simulate scRNA-seq data in which the data generation mechanism is different from our proposed BAMM-SC model. BAMM-SC still achieved higher clustering accuracy than other competing methods. In addition, we compared BAMM-SC with other clustering methods when the number of clusters is different from the true number of cell types. Supplementary Fig. 16 shows that BAMM-SC still achieved the highest ARI in most scenarios.

Other than MNN and CCA, several other approaches have been proposed to correct batch effect across multiple individuals. One straightforward approach is taking one individual as the reference, producing a low-dimensional embedding of it and then projecting the other individuals onto that embedding. To perform low-dimensional embedding, diffusion map^27^ is a tool for nonlinear dimension reduction and has recently been adapted for the visualization of single-cell gene-expression data. In addition, single-cell variational inference (scVI) is a scalable framework for batch correction based on variational inference and stochastic optimization of deep neural networks^28^. The performance of diffusion map and scVI combined with other clustering method was examined, which is worse than MNN and CCA in the three synthetic datasets (possibly due to unmet model underlying assumptions). We will explore more emerging methods in our future work.

There are several limitations of BAMM-SC. First, we filtered out genes with excessive zeros from the analysis under the assumption that lowly-expressed genes do not contribute much to clustering. This may be problematic for rare cell type identification. Second, we do not explicitly model a zero-inflation pattern, which may or may not affect clustering accuracy. A refined model that can handle inflated zeros can be further developed with a balance between computational complexity and model flexibility. Third, in our model, we assume that each cell belongs to one distinct cluster. The posterior probability measures the clustering uncertainty, which cannot be directly interpreted as a quantification of cell cycle or developmental stage. Finally, although our supervised strategy is proven to work for large datasets efficiently, it may potentially miss some rare clusters.

Our method has the potential to be extended to perform trajectory analysis^29,30^, and accounts for both individual and batch level heterogeneity (e.g., two individuals spread evenly across two 10x chips in a properly blocked design) by adding another level of structure. In addition, the model parameters can be used for downstream differential gene expression analysis or construct cell-type specific biomarker panels. These interesting directions are beyond the scope of this paper and will be studied in future papers. Additionally, unlike the traditional way of analyzing scRNA-seq data, BAMM-SC can be also used with batch effect correction. As shown in Supplementary Fig. 17, we ran BAMM-SC on the mouse lung dataset first and extracted cells in cluster 4. Then we applied CCA (implemented in Seurat) on this specific cluster of cells and replotted the t-SNE plot. From Supplementary Fig. 17e, cells from different samples are superimposed on each other, suggesting that most batch effect has been removed. In practice, we recommend using BAMM-SC for clustering raw count data and then use other methods, such as MNN and CCA, to remove batch effect for each individual cell type if needed.

We have applied BAMM-SC to simulated datasets and three in-house synthetic datasets to showcase its performance on different tissue types and species. With the increased popularity of population-based scRNA-seq studies, BAMM-SC will become a powerful tool for elucidating single cell level transcriptomic heterogeneity from population-based studies and a complementary approach to existing clustering methods.

## Methods

### Statistical model

We propose a Bayesian hierarchical Dirichlet multinomial mixture model to explicitly characterize different sources of variability in population scale scRNA-seq data. Specifically, let $x_{ijl}$ represent the number of unique UMIs for gene *i* in cell *j* from individual *l* ($1 \le i \le G$, $1 \le j \le C_l$, $1 \le l \le L$). Here, *G*, *C*_*l*_, and *L* denote the total number of genes, cells (in individual *l*), and individuals, respectively. Our goal is to perform simultaneous clustering for cells from all *L* individuals. We assume that within each individual, all single cells consist of *K* distinct cell types. Cell type clusters are discrete, and each cell belongs to one cell type exclusively. Here, *K* is predefined according to prior biological knowledge, or will be estimated from the data, and *K* is the same among all *L* individuals.

Assume that ${\boldsymbol{x}}_{ \cdot {\boldsymbol{jl}}} = (x_{1jl},x_{2jl}, \ldots ,x_{Gjl})$, the gene expression for cell *j* in individual *l*, follows a multinomial distribution multi $\left( {T_{jl},{\boldsymbol{p}}_{ \cdot {\boldsymbol{jl}}}} \right).$ Here, $T_{jl} = \mathop {\sum }\limits_{i = 1}^G x_{ijl}$ is the total number of UMIs, ${\boldsymbol{p}}_{ \cdot {\boldsymbol{jl}}} = (p_{1jl},p_{2jl}, \ldots ,p_{Gjl})$ is the probability vector for gene expression with $\mathop {\sum }\limits_{i = 1}^G p_{ijl} = 1$, (where larger $p_{ijl}$ is associated with more UMI counts $x_{ijl}$). In addition, let $z_{jl} \in \{ 1,2, \ldots ,K\}$ represent the cell type label for cell *j* in individual *l*, where $z_{jl} = k$ indicates that cell *j* in individual *l* belongs to cell type *k*. Cells of the same cell type share a similar gene-expression pattern. If cell *j* in individual *l* belongs to cell type *k* ($z_{jl} = k$), we assume that ${\boldsymbol{p}}_{ \cdot {\boldsymbol{jl}}}$ follows a cell-type specific Dirichlet prior Dir$\left( {{\boldsymbol{\alpha }}_{ \cdot {\boldsymbol{lk}}}} \right)$, where ${\boldsymbol{\alpha }}_{ \cdot {\boldsymbol{lk}}} = \left( {\alpha _{1lk},\alpha _{2lk}, \ldots ,\alpha _{Glk}} \right)$ is the Dirichlet prior parameter for cell type *k* in individual *l*.

1 $$P\left( {{\boldsymbol{p}}_{.{\boldsymbol{jl}}}|z_{jl} = k,{\boldsymbol{\alpha }}_{ \cdot {\boldsymbol{lk}}}} \right) = \frac{1}{{B({\boldsymbol{\alpha }}_{ \cdot {\boldsymbol{lk}}})}}p_{1jl}^{\alpha _{1lk} - 1}p_{2jl}^{\alpha _{2lk} - 1} \ldots p_{Gjl}^{\alpha _{Glk} - 1},$$

where $B(\alpha _{ \cdot lk})$ is Beta function with parameter $\alpha _{ \cdot lk} = \left( {\alpha _{1lk},\alpha _{2lk}, \ldots ,\alpha _{Glk}} \right)$. Then after integrating $p_{ \cdot jl}$ out, we have:

2 $$P\left( {{\boldsymbol{x}}_{ \cdot {\boldsymbol{jl}}}|z_{jl} = k,{\boldsymbol{\alpha }}_{ \cdot {\boldsymbol{lk}}}} \right) = \frac{{T_{jl}!}}{{\mathop {\prod }\nolimits_{i = 1}^G x_{ijl}!}}\left( {\mathop {\prod }\limits_{i = 1}^G \frac{{{\mathrm{\Gamma }}(x_{ijl} + \alpha _{ilk})}}{{{\mathrm{\Gamma }}(\alpha _{ilk})}}} \right)\frac{{{\mathrm{\Gamma }}(|{\boldsymbol{\alpha }}_{ \cdot {\boldsymbol{lk}}}|)}}{{{\mathrm{\Gamma }}(T_{jl} + |{\boldsymbol{\alpha }}_{ \cdot {\boldsymbol{lk}}}|)}},$$

where $\left| {{\boldsymbol{\alpha }}_{ \cdot {\boldsymbol{lk}}}} \right| = \mathop {\sum }\limits_{i = 1}^G \alpha _{ilk}$. The joint distribution of ${\boldsymbol{x}}_{ \cdot {\boldsymbol{jl}}}$ and $z_{jl}$ is

3 $$P\left( {{\boldsymbol{x}}_{ \cdot {\boldsymbol{jl}}},z_{jl}{\mathrm{|}}{\boldsymbol{\alpha }}_{ \cdot {\boldsymbol{l}} \cdot }} \right) = \frac{{T_{jl}!}}{{\mathop {\prod }\nolimits_{i = 1}^G x_{ijl}!}}\mathop {\sum }\limits_{k = 1}^K I(z_{jl} = k)\left( {\mathop {\prod }\limits_{i = 1}^G \frac{{{\mathrm{\Gamma }}(x_{ijl} + \alpha _{ilk})}}{{{\mathrm{\Gamma }}(\alpha _{ilk})}}} \right)\frac{{{\mathrm{\Gamma }}(|{\boldsymbol{\alpha }}_{ \cdot {\boldsymbol{lk}}}|)}}{{{\mathrm{\Gamma }}\left( {T_{jl} + \left| {{\boldsymbol{\alpha }}_{ \cdot \cdot {\boldsymbol{k}}}} \right|} \right)}}.$$

We further assume that all *C*_*l*_ cells in individual *l* are independent, then the joint distribution for all cells in individual *l* is

4 $$P\left( {{\boldsymbol{x}}_{ \cdot \cdot {\boldsymbol{l}}},{\boldsymbol{z}}_{ \cdot {\boldsymbol{l}}}|{\boldsymbol{\alpha }}_{ \cdot {\boldsymbol{l}} \cdot }} \right) = \mathop {\prod }\limits_{j = 1}^{C_l} P\left( {{\boldsymbol{x}}_{ \cdot {\boldsymbol{jl}}},z_{jl}{\mathrm{|}}{\boldsymbol{\alpha }}_{ \cdot {\boldsymbol{l}} \cdot }} \right).$$

Finally, we assume that all *L* individuals are independent, then the overall joint distribution for all cells across all individuals becomes

5 $$P\left( {{\boldsymbol{x}}_{ \cdot \cdot \cdot },{\boldsymbol{z}}_{ \cdot \cdot }|{\boldsymbol{\alpha }}_{ \cdot \cdot \cdot }} \right) = \mathop {\prod }\limits_{l = 1}^L P\left( {{\boldsymbol{x}}_{ \cdot \cdot {\boldsymbol{l}}},{\boldsymbol{z}}_{ \cdot {\boldsymbol{l}}}|{\boldsymbol{\alpha }}_{ \cdot {\boldsymbol{l}}\, \cdot }} \right) \\ \propto \mathop {\prod }\limits_{l = 1}^L \mathop {\prod }\limits_{j = 1}^{C_l} \left\{ {\mathop {\sum }\limits_{k = 1}^K I\left( {z_{jl} = k} \right)\left( {\mathop {\prod }\limits_{i = 1}^G \frac{{{\mathrm{\Gamma }}\left( {x_{ijl} + \alpha _{ilk}} \right)}}{{{\mathrm{\Gamma }}\left( {\alpha _{ilk}} \right)}}} \right)\frac{{{\mathrm{\Gamma }}\left( {\left| {{\boldsymbol{\alpha }}_{ \cdot {\boldsymbol{lk}}}} \right|} \right)}}{{{\mathrm{\Gamma }}\left( {T_{jl} + \left| {{\boldsymbol{\alpha }}_{ \cdot {\boldsymbol{lk}}}} \right|} \right)}}} \right\}.$$

In this model, the two sets of parameters of interest are ${\boldsymbol{z}}_{ \cdot \cdot } = \left\{ {z_{jl}} \right\}_{1 \le j \le C_l,1 \le l \le L}$, the cell type label for cell *j* in individual *l*, and ${\boldsymbol{\alpha }}_{ \cdot \cdot \cdot } = \left\{ {\alpha _{ilk}} \right\}_{1 \le i \le G,1 \le l \le L,1 \le k \le K}$, the Dirichlet parameters for gene *i* in cell type *k* in individual *l*. We adopt a full Bayesian approach and use Gibbs sampler to estimate the posterior distributions. Specifically, the joint posterior distribution for ${\boldsymbol{z}}_{ \cdot \cdot }$ and ${\boldsymbol{\alpha }}_{ \cdot \cdot \cdot }$ are

6 $$P\left( {{\boldsymbol{z}}_{ \cdot \cdot },{\boldsymbol{\alpha }}_{ \cdot \cdot \cdot }|{\boldsymbol{x}}_{ \cdot \cdot \cdot }} \right) \propto P\left( {{\boldsymbol{x}}_{ \cdot \cdot \cdot },{\boldsymbol{z}}_{ \cdot \cdot }|{\boldsymbol{\alpha }}_{ \cdot \cdot \cdot }} \right) \times Prior\left( {{\boldsymbol{\alpha }}_{ \cdot \cdot \cdot }} \right).$$

Since all *α*’s are strictly positive, we propose a log-normal distribution as the prior distribution for $\alpha _{ilk}$. We assume that for gene *i* in cell type *k*, $\alpha _{ilk}$ from all *L* individuals share the same prior distribution LN $(\mu _{ik},\sigma _{ik}^2)$, that is

7 $${\mathrm{Prior}}\left( {{\boldsymbol{\alpha }}_{{\boldsymbol{i}} \cdot {\boldsymbol{k}}}} \right) = \mathop {\prod }\limits_{l = 1}^L \frac{1}{{\alpha _{ilk}\sqrt {2\pi \sigma _{ik}^2} }}\exp \left\{ { - \frac{{\left( {\log \alpha _{ilk} - \mu _{ik}} \right)^2}}{{2\sigma _{ik}^2}}} \right\}.$$

Here, $\mu _{ik}$ can be estimated by the mean of $\alpha _{ilk}$'s: $\hat \mu _{ik} = \frac{1}{L}\mathop {\sum }\limits_{l = 1}^L {\mathrm{log}}(\alpha _{ilk})$. Estimation of $\sigma _{ik}^2$ can be challenging due to limited number of individuals. We can assume all $\sigma _{ik}^2$’s follow a hyper-prior: Gamma distribution Gamma$(a_k,b_k)$, and use information across all genes to estimate variance. In addition, we assume a noninformative prior for $\mu _{ik}$’s. Taken all together, we have the full posterior distribution as follows:

8 $$P\left( {{\boldsymbol{z}}_{ \cdot \cdot },{\boldsymbol{\alpha }}_{ \cdot \cdot \cdot }|{\boldsymbol{x}}_{ \cdot \cdot \cdot }} \right) \propto P\left( {{\boldsymbol{x}}_{ \cdot \cdot \cdot },{\boldsymbol{z}}_{ \cdot \cdot }|{\boldsymbol{\alpha }}_{ \cdot \cdot \cdot }} \right) \times \mathop {\prod }\limits_{k = 1}^K \mathop {\prod }\limits_{i = 1}^G {\mathrm{prior}}\left( {{\boldsymbol{\alpha }}_{{\boldsymbol{i}} \cdot {\boldsymbol{k}}}} \right) \times \mathop {\prod }\limits_{k = 1}^K {\mathrm{prior}}\left( {{\boldsymbol{\mu }}_{ \cdot {\boldsymbol{k}}}} \right) \times \mathop {\prod }\limits_{k = 1}^K {\mathrm{prior}}\left( {{\boldsymbol{\sigma }}_{ \cdot {\boldsymbol{k}}}^2} \right).$$

We use Gibbs sample to iteratively update $\alpha _{ilk}$ and $z_{jl}$. Details can be found in Supplementary Methods.

### Classification and computational acceleration

To further improve the computational efficiency, we provide a supervised option in BAMM-SC. Specifically, for very large-scale dataset, we use BAMM-SC to train a prediction model using a subset of cells from each individual and predict the clustering labels for the rest of cells. First, we randomly select a subset of cells from each individual and applied BAMM-SC on these selected cells. The estimate of $\alpha _{ilk}$ is computed as the average after deletion of the first 100 (default) iterations as burn-in. We then predict the cell type labels for other cells with realization of parameters: $\hat \Theta = \left( {{\hat{\boldsymbol{\alpha }}}_{ \cdot 1 \cdot }, \ldots ,{\hat{\boldsymbol{\alpha }}}_{ \cdot {\boldsymbol{L}} \cdot },{\hat{\boldsymbol{\pi }}}_1, \ldots ,{\hat{\boldsymbol{\pi }}}_{\boldsymbol{L}}} \right)$.

9 $$P\left( {z_{jl} = k{\mathrm{|}}{\boldsymbol{x}}_{{\boldsymbol{jl}}},\hat \Theta } \right) = \frac{{\left( {\mathop {\prod }\nolimits_{i = 1}^G \frac{{{\mathrm{\Gamma }}\left( {x_{ijl} + \hat \alpha _{ilk}} \right)}}{{{\mathrm{\Gamma }}\left( {\hat \alpha _{ilk}} \right)}}} \right)\frac{{{\mathrm{\Gamma }}\left( {|{\hat{\boldsymbol{\alpha }}}_{ \cdot {\boldsymbol{lk}}}|} \right)}}{{{\mathrm{\Gamma }}\left( {T_j + |{\hat{\boldsymbol{\alpha }}}_{ \cdot {\boldsymbol{lk}}}|} \right)}}\hat \pi _{lk}}}{{\mathop {\sum }\nolimits_{k = 1}^K \left( {\mathop {\prod }\nolimits_{i = 1}^G \frac{{{\mathrm{\Gamma }}\left( {x_{ij} + \hat \alpha _{ilk}} \right)}}{{{\mathrm{\Gamma }}\left( {\hat \alpha _{ilk}} \right)}}} \right)\frac{{{\mathrm{\Gamma }}\left( {|{\hat{\boldsymbol{\alpha }}}_{ \cdot {\boldsymbol{lk}}}|} \right)}}{{{\mathrm{\Gamma }}\left( {T_j + |{\hat{\boldsymbol{\alpha }}}_{ \cdot {\boldsymbol{lk}}}|} \right)}}\hat \pi _{lk}}}.$$

This approach can substantially reduce the computational cost for very large-scale datasets while maintaining the accuracy as shown in Supplementary Fig. 14.

### Single-cell sequencing library construction

10× Genomics Chromium system, which is a microfluidics platform based on Gel bead in EMulsion (GEM) technology, was used for generating real test datasets. Cells mixed with reverse transcription reagents were loaded into the Chromium instrument. This instrument separated cells into minireaction partitions formed by oil microdroplets, each containing a gel bead and a cell, known as GEMs. GEMs contain a gel bead, scaffold for an oligonucleotide that is composed of an oligo-dT section for priming reverse transcription, and barcodes for each cell and each transcript as described. GEM generation takes place in a multiple-channel microfluidic chip that encapsulates single-gel beads. Reverse transcription takes place inside each droplet. Approximately, 1000-fold excess of partitions compared to cells ensured low capture of duplicate cells. The reaction mixture/emulsion was removed from the Chromium instrument, and reverse transcription was performed. The emulsion was then broken using a recovery agent, and following Dynabead and SPRI clean up cDNAs were amplified by PCR (C1000, Bio-Rad). cDNAs were sheared (Covaris) into ~200 bp length. DNA fragment ends were repaired, A-tailed and adapters ligated. The library was quantified using KAPA Universal Library Quantification Kit KK4824 and further characterized for cDNA length on a Bioanalyzer using a High Sensitivity DNA kit. All sequencing experiments were conducted using Illumina NextSeq 500 in the Genomics Sequencing Core at the University of Pittsburgh.

### Data description

Human PBMC dataset: Under a protocol approved by the University of Pittsburgh Institutional Review Board, peripheral blood was obtained from healthy donors by venipuncture. Each subject gave written informed consent. PBMC were isolated from whole blood by density gradient centrifugation using Ficoll–Hypaque. PBMC were then counted and resuspended in phosphate buffered saline with 0.04% bovinue serum albumin, and were processed through the Chromium 10× Controller according to the manufacturers’ instructions, targeting a recovery of ~2000 cells. The following steps were all performed under the aforementioned protocol developed by 10× Genomics.

Human skin dataset: Skin samples were obtained by performing 3 mm punch biopsies from the dorsal midforearm of healthy control subjects after informed consent under a protocol approved by the University of Pittsburgh Institutional Review Board. Skin for scRNA-seq was digested enzymatically (Miltenyi Biotec Whole Skin Dissociation Kit, human) for 2 h and further dispersed using the Miltenyi gentleMACS Octo Dissociator. The resulting cell suspension was filtered through 70 micron cell strainers twice and re-suspended in phosphate-buffered saline containing 0.04% bovine serum albumin. Cells from biopsies were mixed with reverse transcription reagents then loaded into the Chromium instrument (10× Genomics). Totally, ~2600–4300 cells were loaded into the instrument to obtain data on ~1100–1800 cells, anticipating a multiplet rate of ~1.2% of partitions. The following steps were all performed under the aforementioned protocol developed by 10× Genomics.

Mouse lung dataset: Lung single cell suspension from naïve and infected C57BL/6 mice were subject to scRNA-seq library preparation protocol. Briefly, left lobs of both naïve and infected mice were removed and digested by Collagenase/DNase to obtain single-cell suspension. Mononuclear cells after filtration with a 40 μM cell strainer were separated into minireaction partitions or GEMs formed by oil microdroplets, each containing a gel bead and a cell, by the Chromium instrument (10× Genomics). The reaction mixture/emulsion with captured and barcoded mRNAs were removed from the Chromium instrument followed by reverse transcription. The cDNA samples were fragmented and amplified using the Nextera XT kit (Illumina). The following steps were all performed under aforementioned the protocol developed by 10× Genomics. We have complied with all relevant ethical regulations for animal research. The animal protocol was approved by the University of Pittsburgh Institutional Animal Care and Use Committee.

### Reporting summary

Further information on experimental design is available in the Nature Research Reporting Summary linked to this article.

## Supplementary information

Supplementary Information

Reporting Summary

## Data Availability

The study uses various publicly available scRNA-seq datasets. Both human PBMC (sample 5) and purified CD19+B cell scRNA-seq data that support the findings of this study are available at https://support.10xgenomics.com/single-cell-gene-expression/datasets. The raw fastq files and preprocessed experimental test datasets (human PBMCs, mouse lung and human skin tissues) have been deposited in the gene expression omnibus (GEO) database under accession number GSE128066. All other relevant data are available upon request.

## Code availability

BAMM-SC, including all source and example code, is freely available as an R package with a detailed tutorial at https://github.com/CHPGenetics/BAMMSC.
