# Supplementary material for: A Bayesian mixture model for clustering droplet-based single-cell transcriptomic data from population studies
Source: Nat Commun. 2019 Apr 9;10:1649. doi: 10.1038/s41467-019-09639-3 (PMC6456731; doi:10.1038/s41467-019-09639-3)
Supplement: Supplementary file 1 — Supplementary Information [file 41467_2019_9639_MOESM1_ESM.pdf]

Supplementary Information for

A Bayesian mixture model for clustering droplet-based single cell transcriptomic data from population studies

Zhe Sun[1, §], Li Chen[2, §], Hongyi Xin[3], Yale Jiang[3, 4], Qianhui Huang[5], Anthony R Cillo[6], Tracy Tabib[7], Jay K Kolls[8], Tullia C Bruno[6, 9], Robert Lafyatis[7], Dario AA Vignali[6, 9, 10], Kong Chen[11], Ying Ding[1, *], Ming Hu[12, *], and Wei Chen[1, 3, *]

[1]Department of Biostatistics, Graduate School of Public Health, University of Pittsburgh, Pittsburgh, Pennsylvania, USA
[2]Department of Health Outcomes Research and Policy, Harrison School of Pharmacy, Auburn University, Auburn, Alabama, USA
[3]Division of Pulmonary Medicine, Department of Pediatrics, Children's Hospital of Pittsburgh of UPMC, University of Pittsburgh, Pittsburgh, Pennsylvania, USA
[4]School of Medicine, Tsinghua University, Beijing, China
[5]Department of Biostatistics, School of Public Health, University of Michigan, Ann Arbor, Michigan, USA
[6]Department of Immunology, School of Medicine, University of Pittsburgh, Pittsburgh, Pennsylvania, USA
[7]Division of Rheumatology and Clinical Immunology, Department of Medicine, School of Medicine, University of Pittsburgh, Pittsburgh, Pennsylvania, USA
[8]School of Medicine, Tulane University, New Orleans, Louisiana, USA
[9]Tumor Microenvironment Center, UPMC Hillman Cancer Center, Pittsburgh, Pennsylvania, USA
[10]Cancer Immunology and Immunotherapy Program, UPMC Hillman Cancer Center, Pittsburgh, Pennsylvania, USA
[11]Division of Pulmonary, Allergy and Critical Care Medicine, Department of Medicine, School of Medicine, University of Pittsburgh, Pittsburgh, Pennsylvania, USA
[12]Department of Quantitative Health Sciences, Lerner Research Institute, Cleveland Clinic Foundation, Cleveland, Ohio, USA

[§] These authors contributed equally to this work.

[*] Co-corresponding authors

Contact: wei.chen@chp.edu or hum@ccf.org or yingding@pitt.edu

Supplementary Figure 1. The t-SNE projection of human PBMC dataset, colored by different sample IDs.

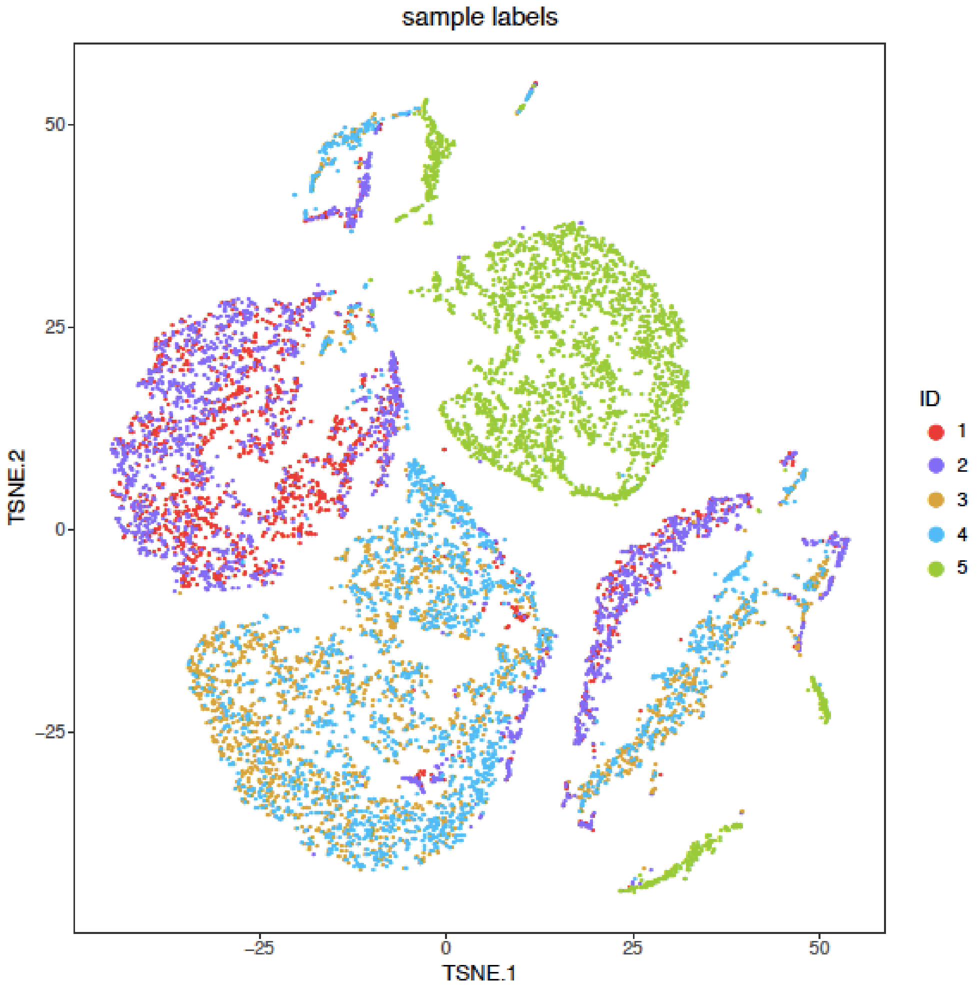

Supplementary Figure 2. The Boxplots of ARI for ten clustering methods across 100 simulations, investigating how different number of clusters (a), sequencing depth (b) and cell-type-specific heterogeneities (c) affect clustering results.

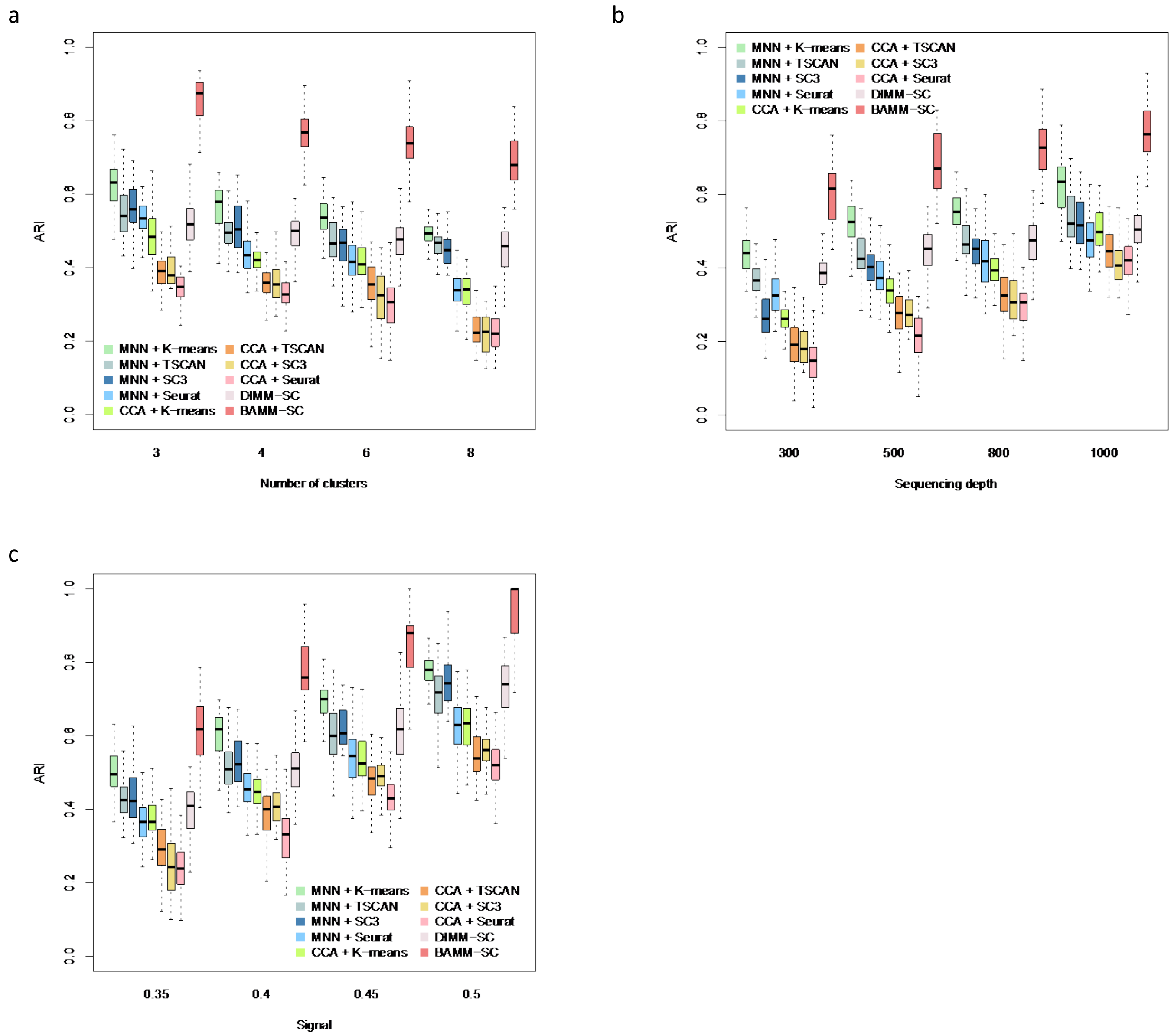

Supplementary Figure 3. The t-SNE projection of cells from human PBMC, colored by different types of PBMCs based on the biological knowledge of cell-type-specific gene markers.

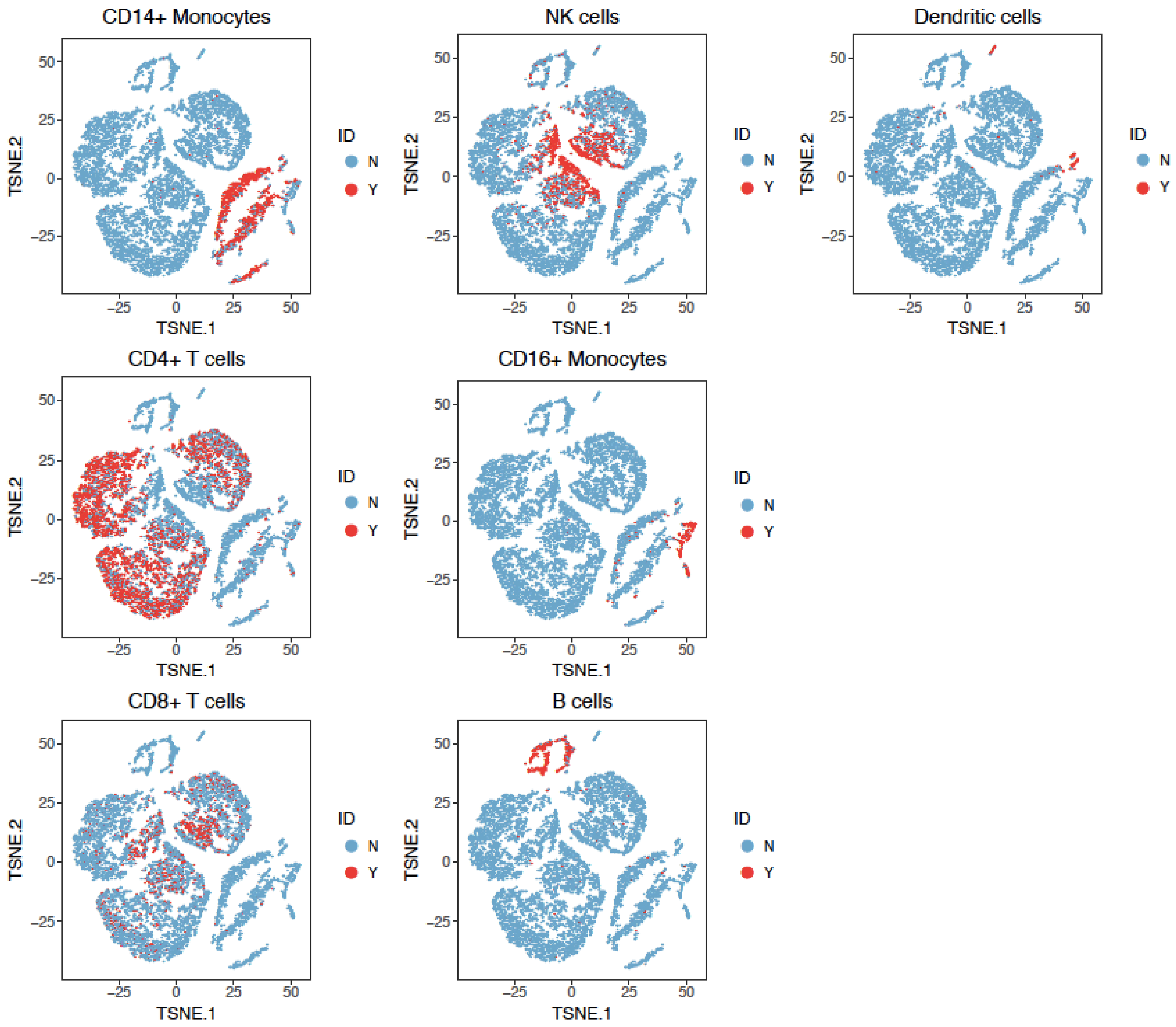

Supplementary Figure 4. The t-SNE projection of cells from human PBMC dataset, colored by the MNN + K-means clustering (a), MNN + TSCAN (b), MNN + SC3 (c), MNN + Seurat (d), CCA + K-means (e), CCA + TSCAN (f), CCA + SC3 (g), CCA + Seurat (h) and DIMM-SC (i) clustering assignment. All clustering labels are from the result with the highest ARI among 10 times analysis.

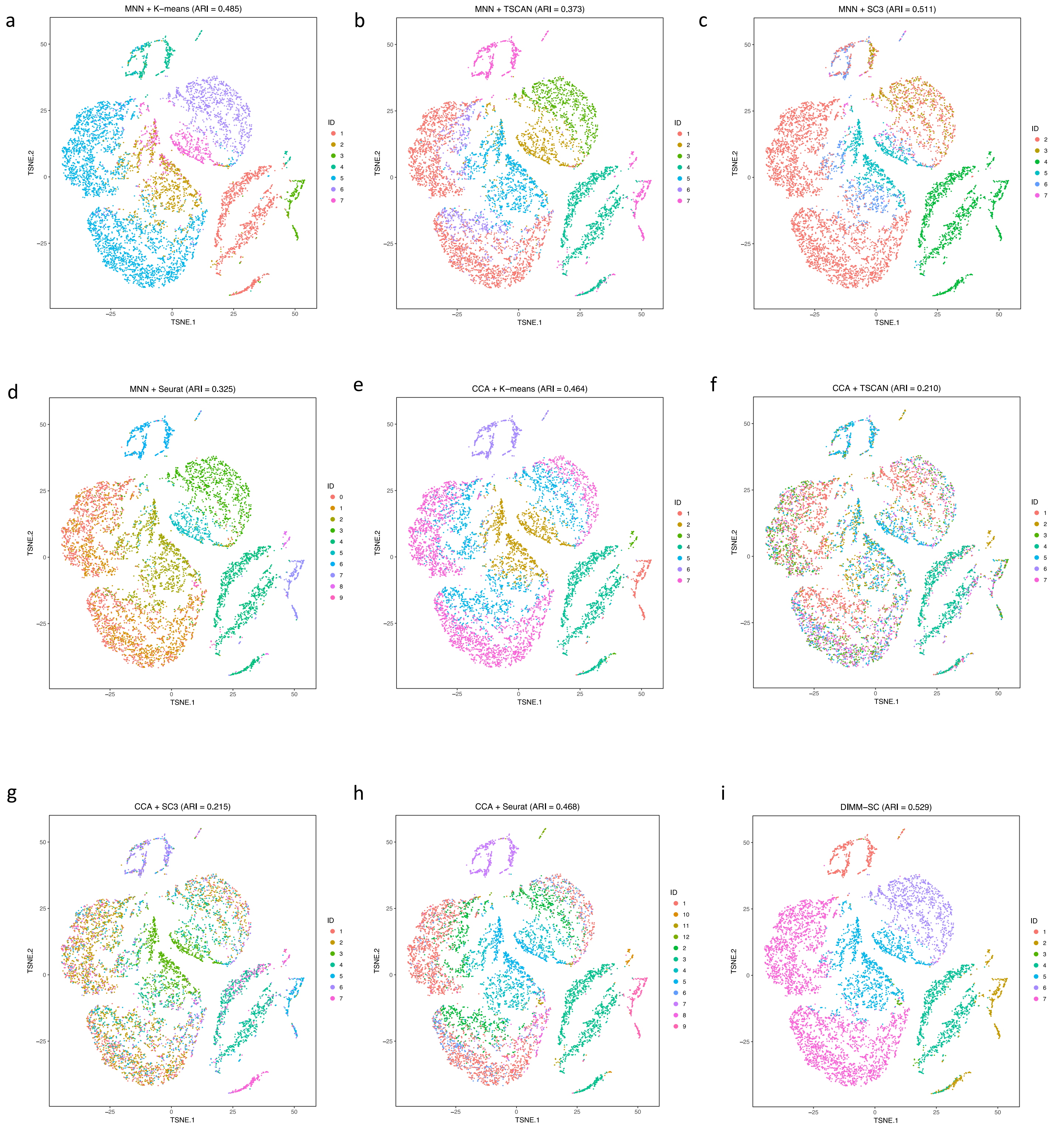

Supplementary Figure 5. The t-SNE projection of mouse lung dataset, colored by different sample IDs.

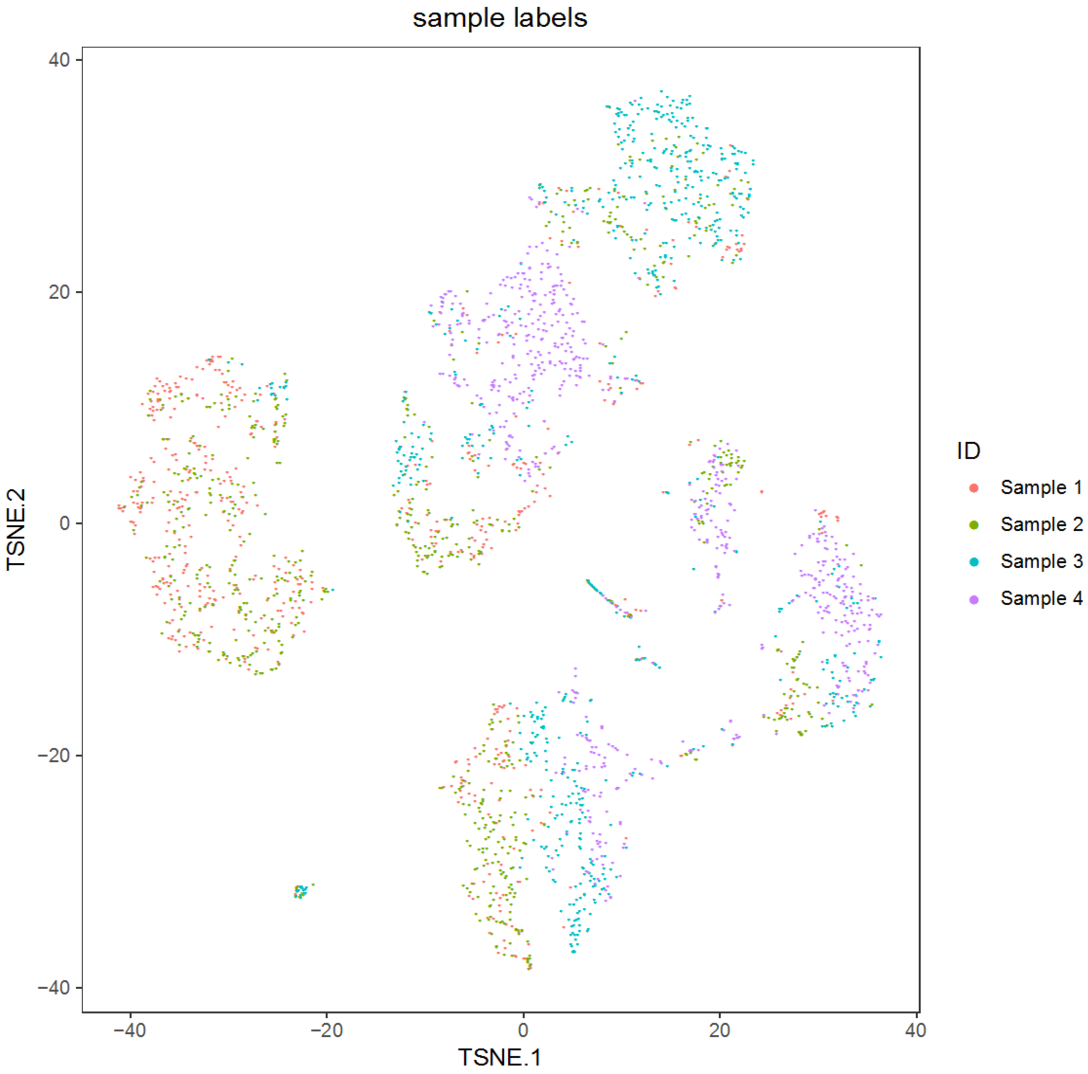

Supplementary Figure 6. The t-SNE projection of cells from mouse lung dataset, colored by different types of PBMCs based on the biological knowledge of cell-type-specific gene markers.

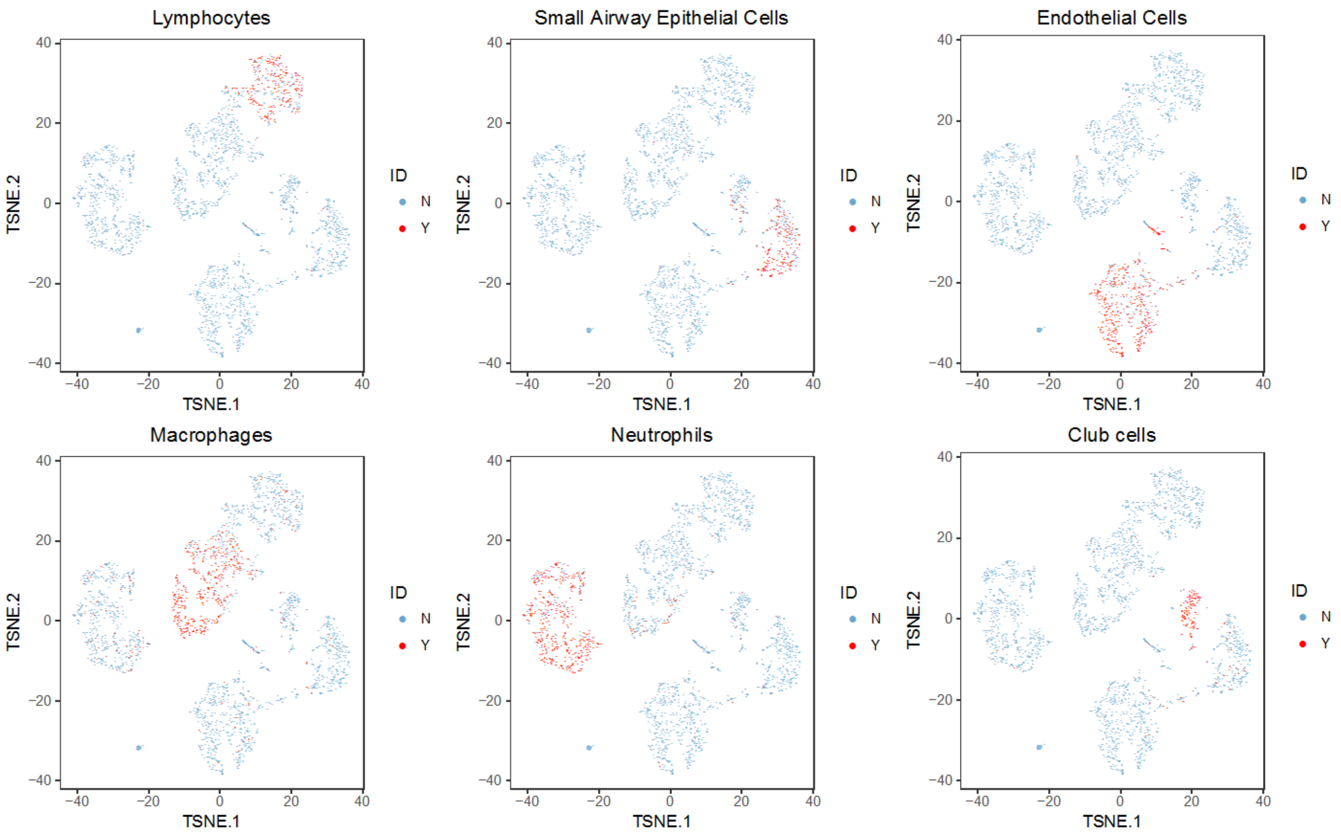

Supplementary Figure 7. The t-SNE projection of cells from mouse lung dataset, colored by the MNN + K-means clustering (a), MNN + TSCAN (b), MNN + SC3 (c), MNN + Seurat (d), CCA + K-means (e), CCA + TSCAN (f), CCA + SC3 (g), CCA + Seurat (h) and DIMM-SC (i) clustering assignment. All clustering labels are from the result with the highest ARI among 10 times analysis.

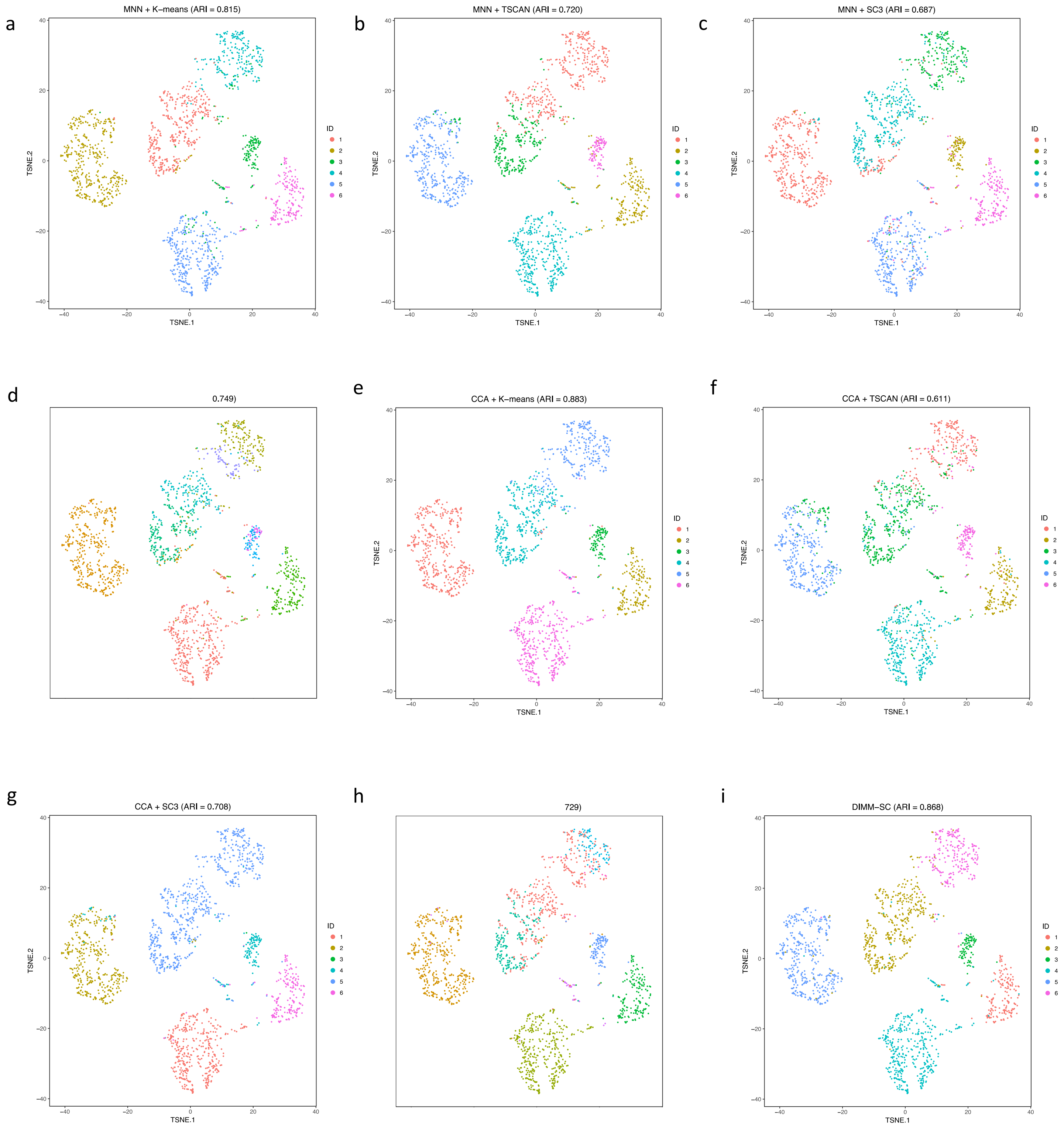

Supplementary Figure 8. Bar plots of proportions of cell types for each individual in mouse lung dataset.

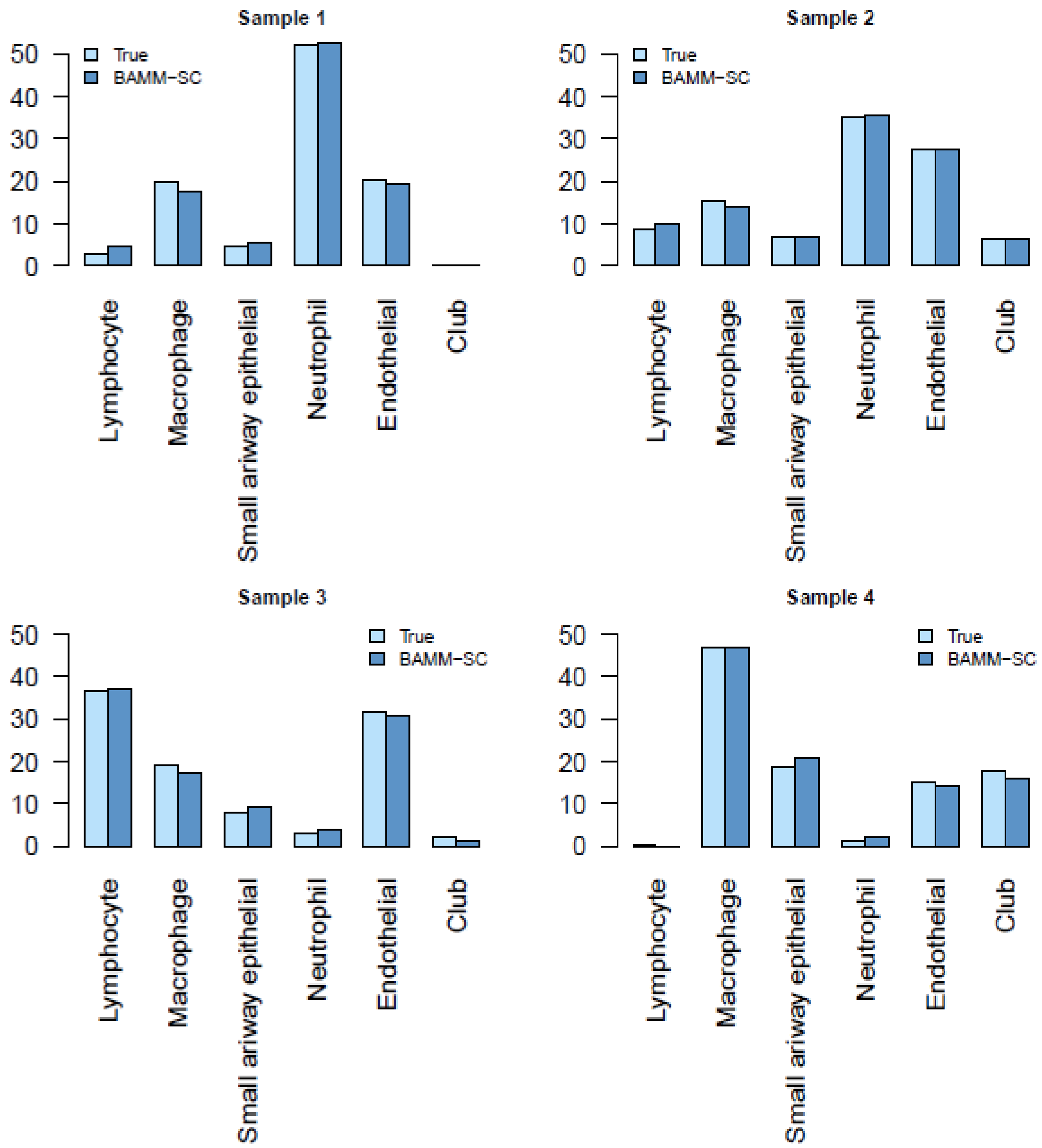

Supplementary Figure 9. The t-SNE projection of cells from human skin dataset, colored by different sample IDs.

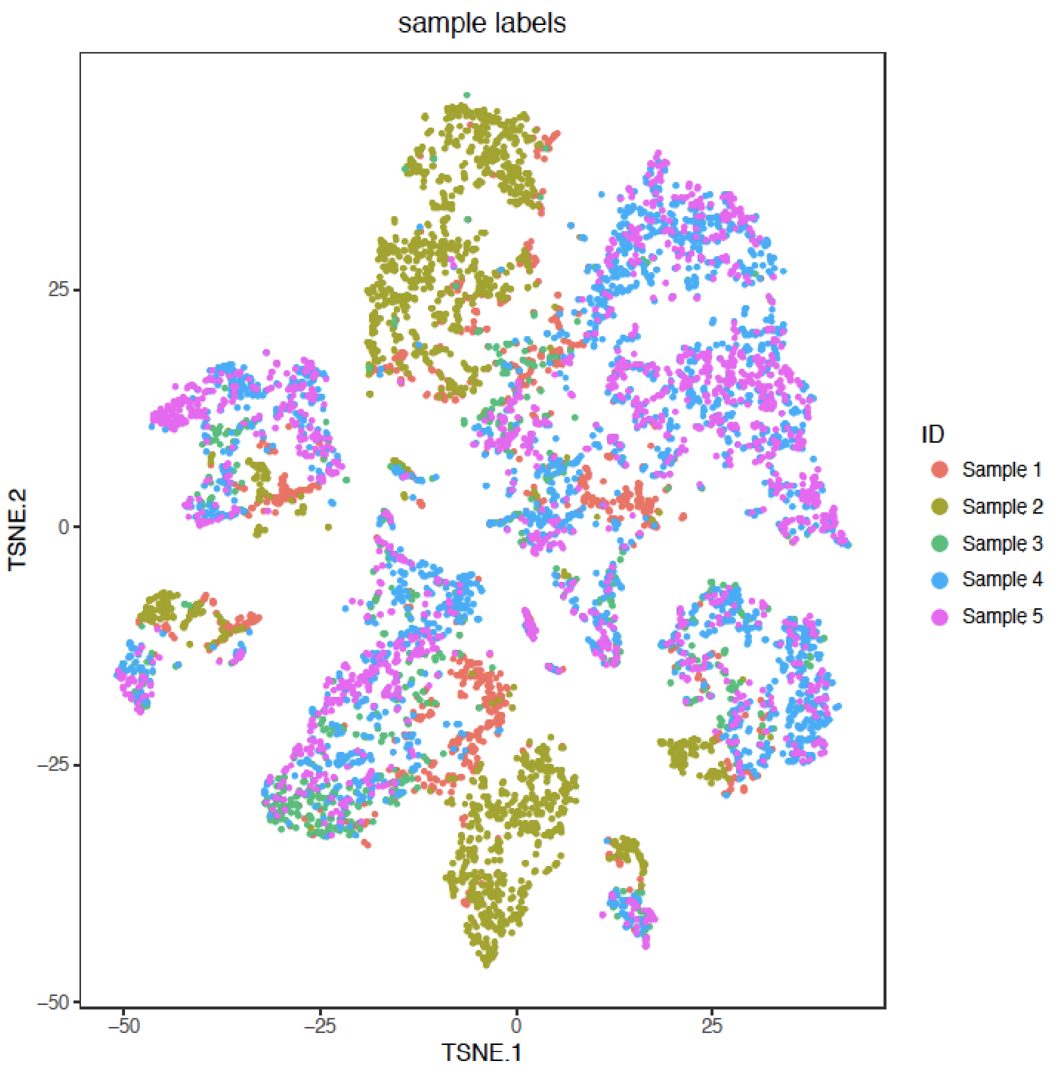

Supplementary Figure 10. The t-SNE projection of cells from human skin dataset, colored by different types of PBMCs based on the biological knowledge of cell-type-specific gene markers.

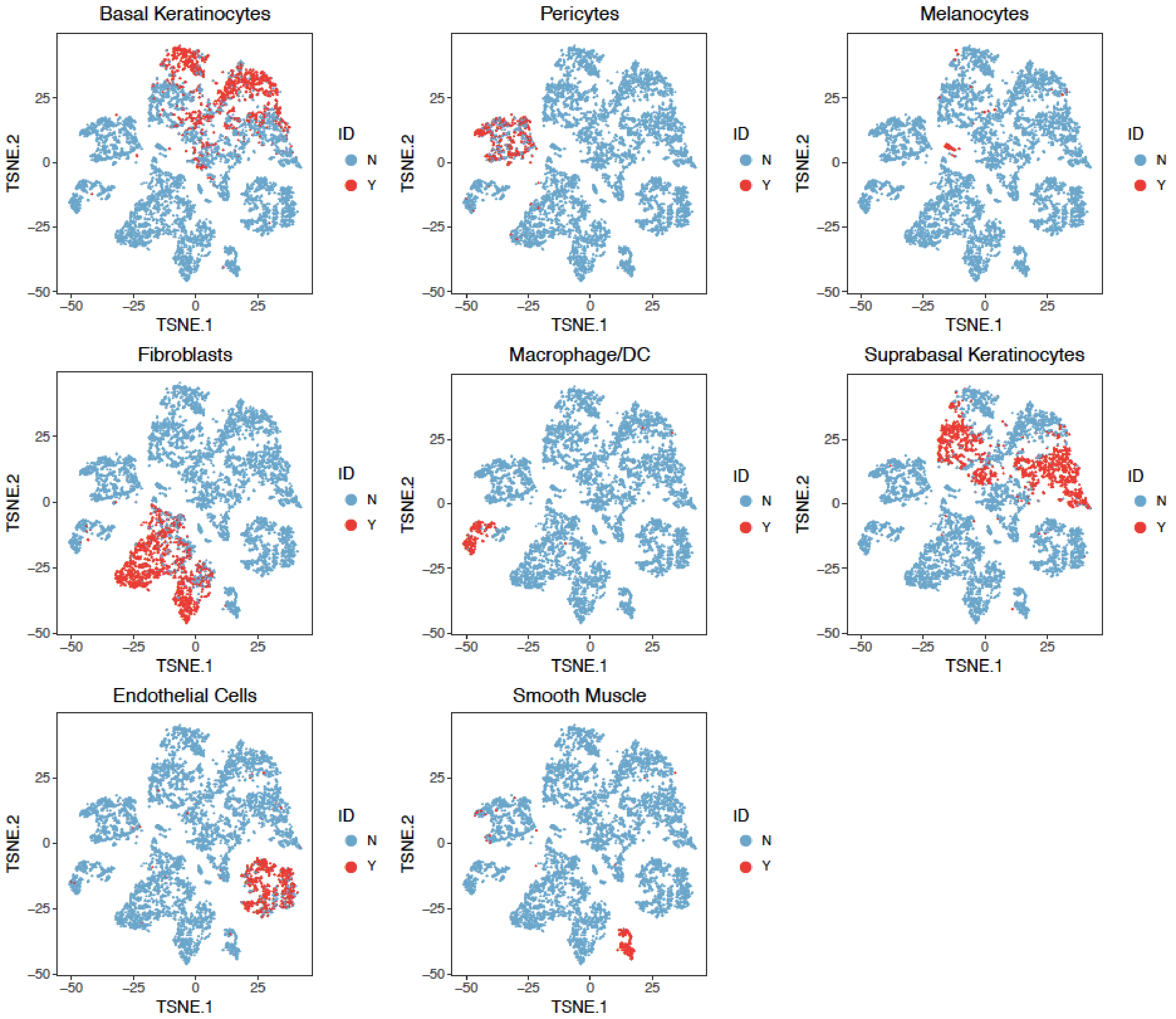

Supplementary Figure 11. The t-SNE projection of cells from human skin dataset, colored by the MNN + K-means clustering (a), MNN + TSCAN (b), MNN + SC3 (c), MNN + Seurat (d), CCA + K-means (e), CCA + TSCAN (f), CCA + SC3 (g), CCA + Seurat (h) and DIMM-SC (i) clustering assignment. All clustering labels are from the result with the highest ARI among 10 times analysis.

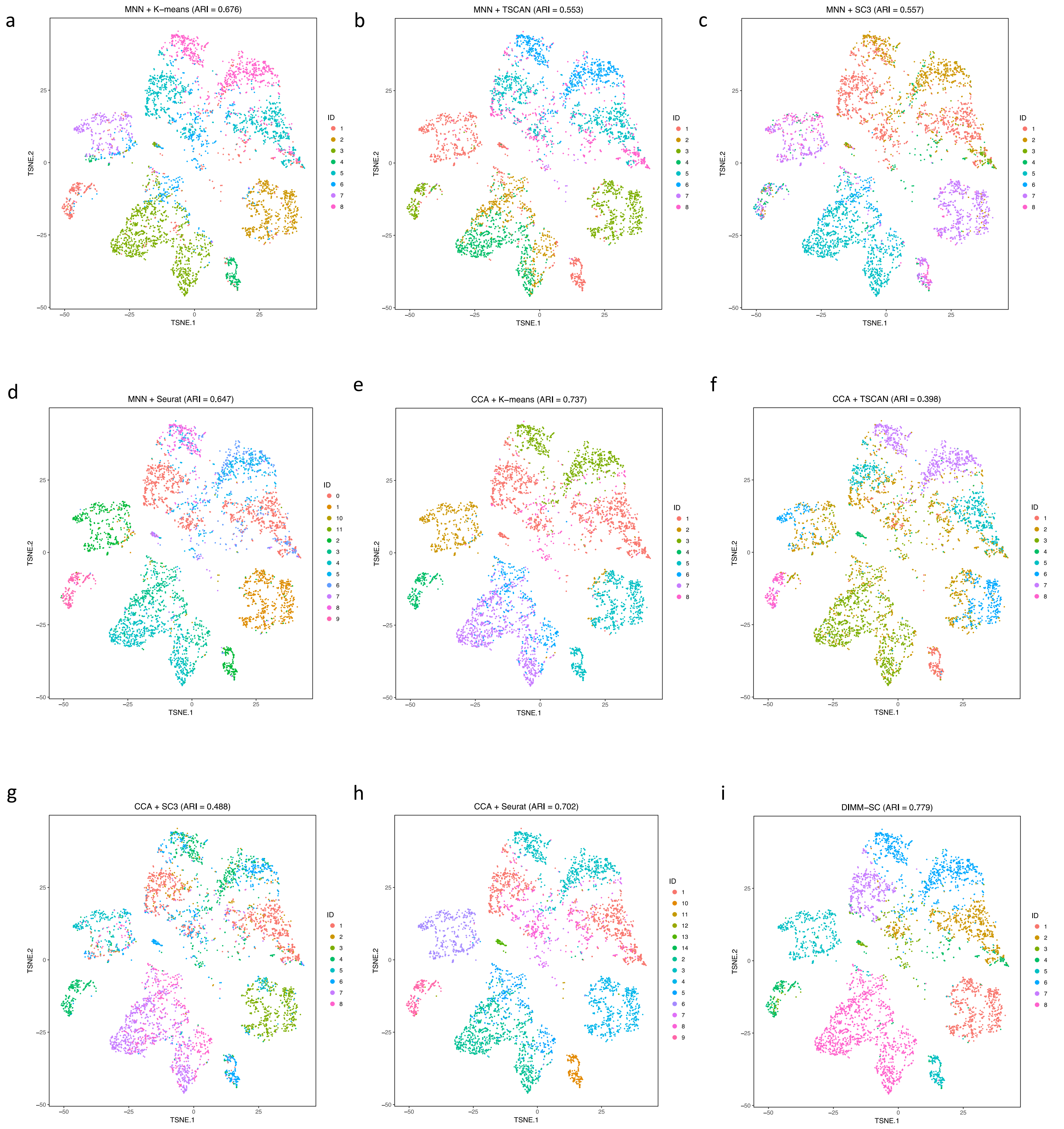

Supplementary Figure 12. Bar plot of cell proportions from flow cytometry and different clustering methods in individual 3 from the human PBMC dataset. All clustering assignments are from the result with the highest ARI among 10 times analysis.

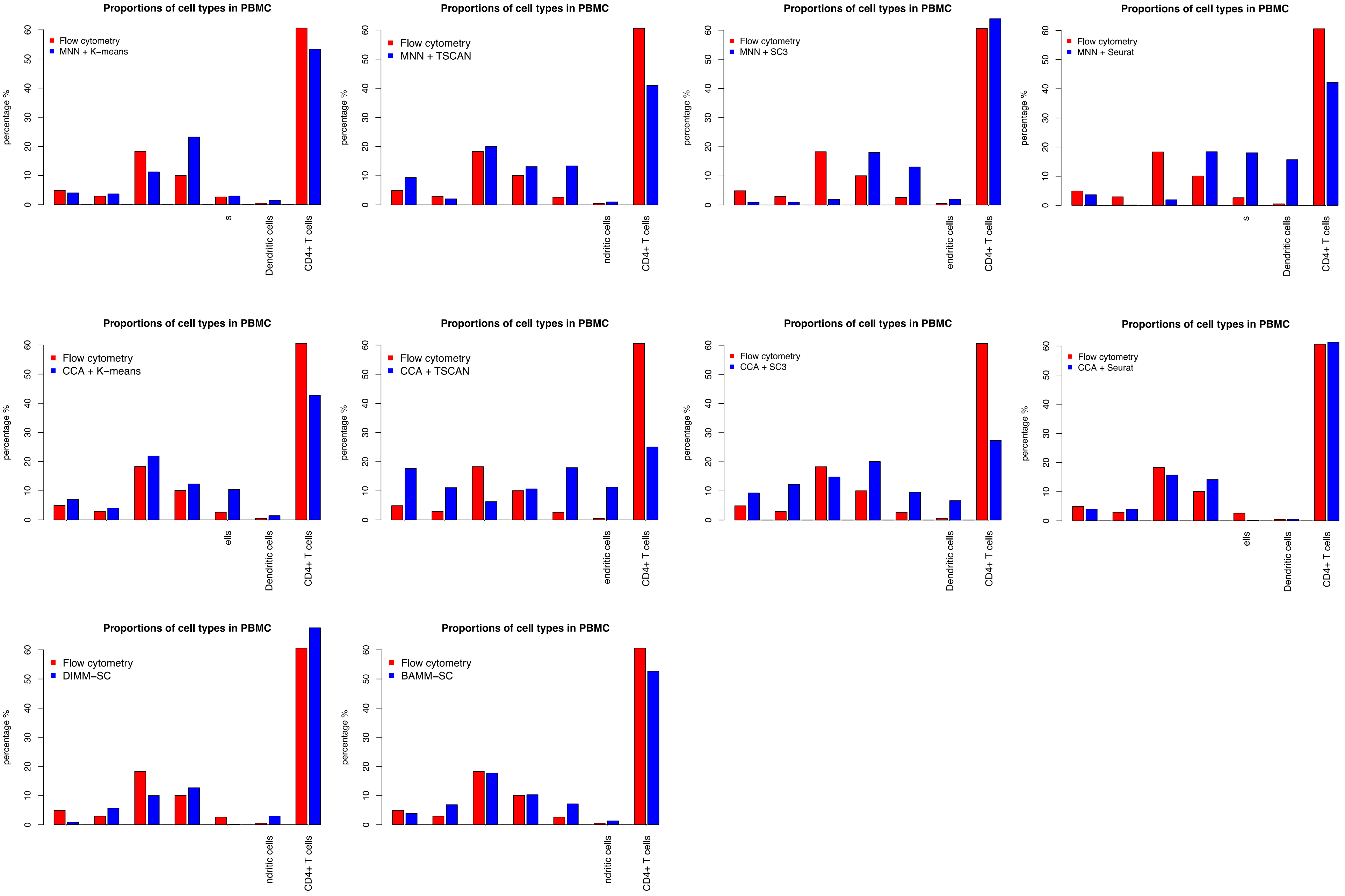

Supplementary Figure 13. The t-SNE projection of human PBMC samples, colored by the approximated truth (a), BAMM-SC clustering results (b) and the illustration of vague cells with the largest posterior probability < 0.95 (c)

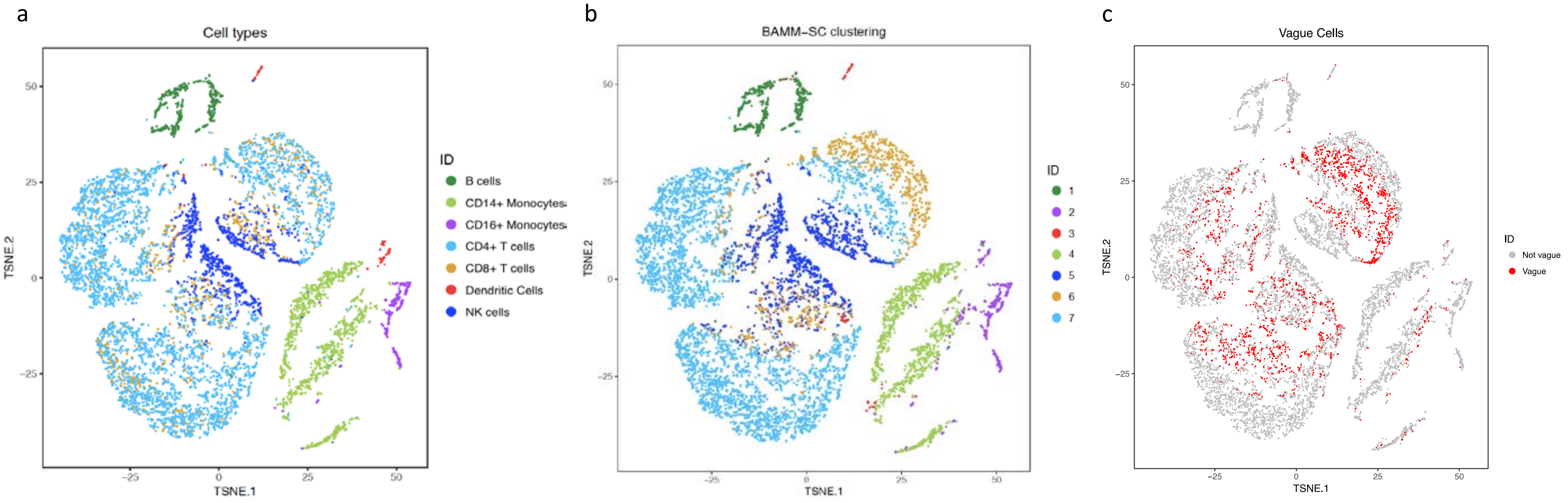

Supplementary Figure 14. The bar plot of computational time for BAMM-SC in simulated dataset with different number of cells in each individual (a), different number of individuals (b), different number of clusters (c), and the bar plot of computational time of different clustering methods in simulated dataset (d). In (d), we set the number of single cells in each individual as 4,000, the number of individuals as 10, and the number of clusters as 4, to benchmark the computational cost of different methods. To be noted, K-means clustering itself is very fast, the process of batch effect correction and calculating dimension reduction representations takes most of the computational time.

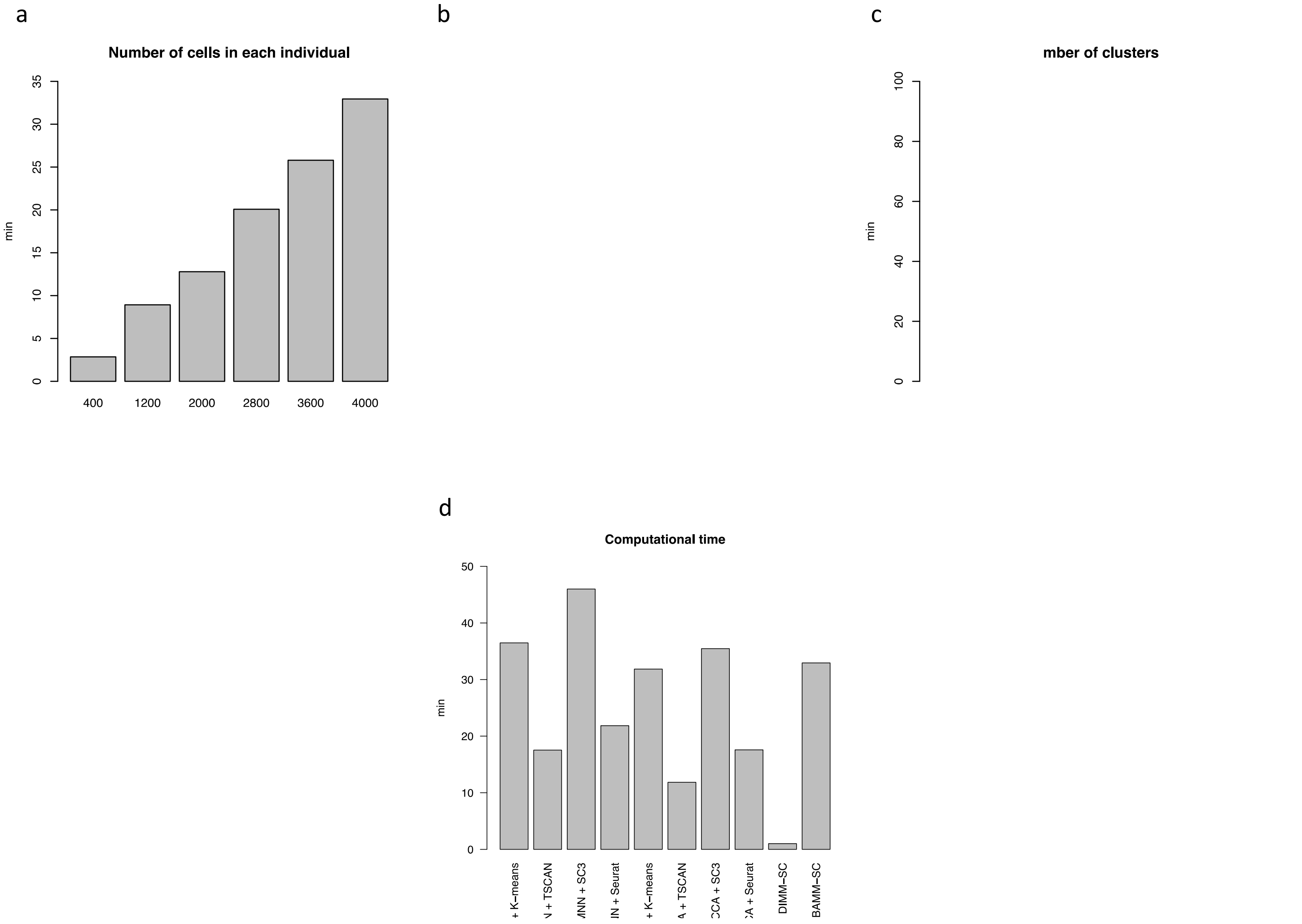

Supplementary Figure 15. The dot plots of AIC and BIC for the final clustering results in the simulated dataset, where the true number of clusters is 4. Blue dots and red dots denote values of BIC and AIC, respectively. Black dots denote ARIs.

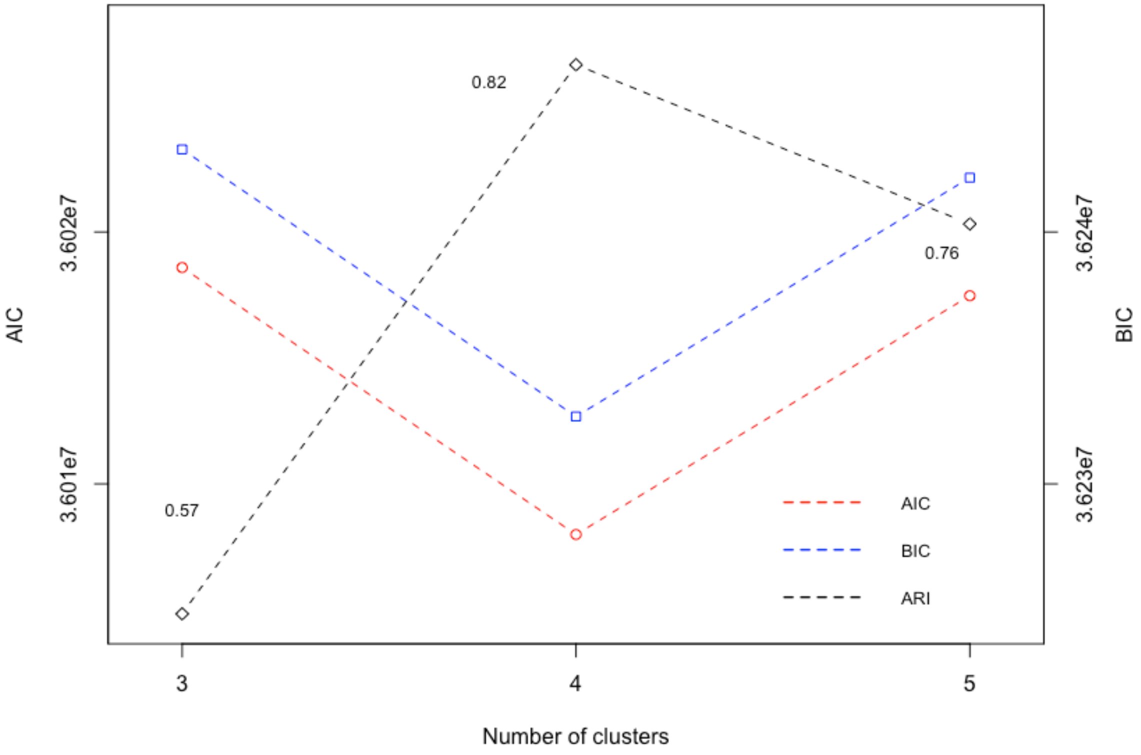

Supplementary Figure 16. The Boxplots of ARI for ten clustering methods across 100 simulations when number of clusters is mis-specified using simulated data (a) and data generated from Splatter (b)

a

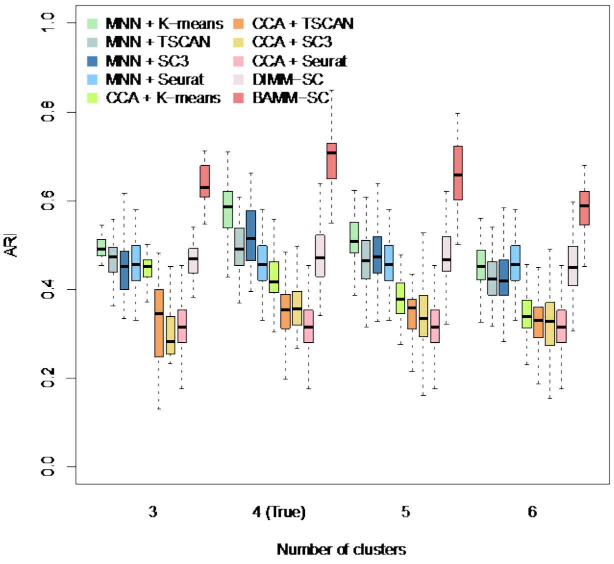

b

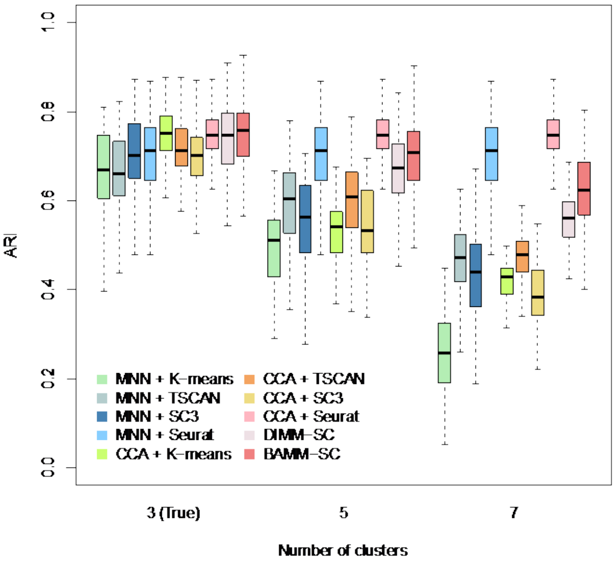

Supplementary Figure 17. The t-SNE projection of cells from mouse lung dataset without batch effect correction, colored by different sample IDs (a) and BAMM-SC clustering assignment (b), the t-SNE projection of cells after CCA batch effect correction, colored by different sample IDs (c) and the clustering assignment (based on the result of BAMM-SC in (b)) (d), and the t-SNE projection of cells in cluster 4 (based on the result of BAMM-SC in (b)) with CCA correction (e)

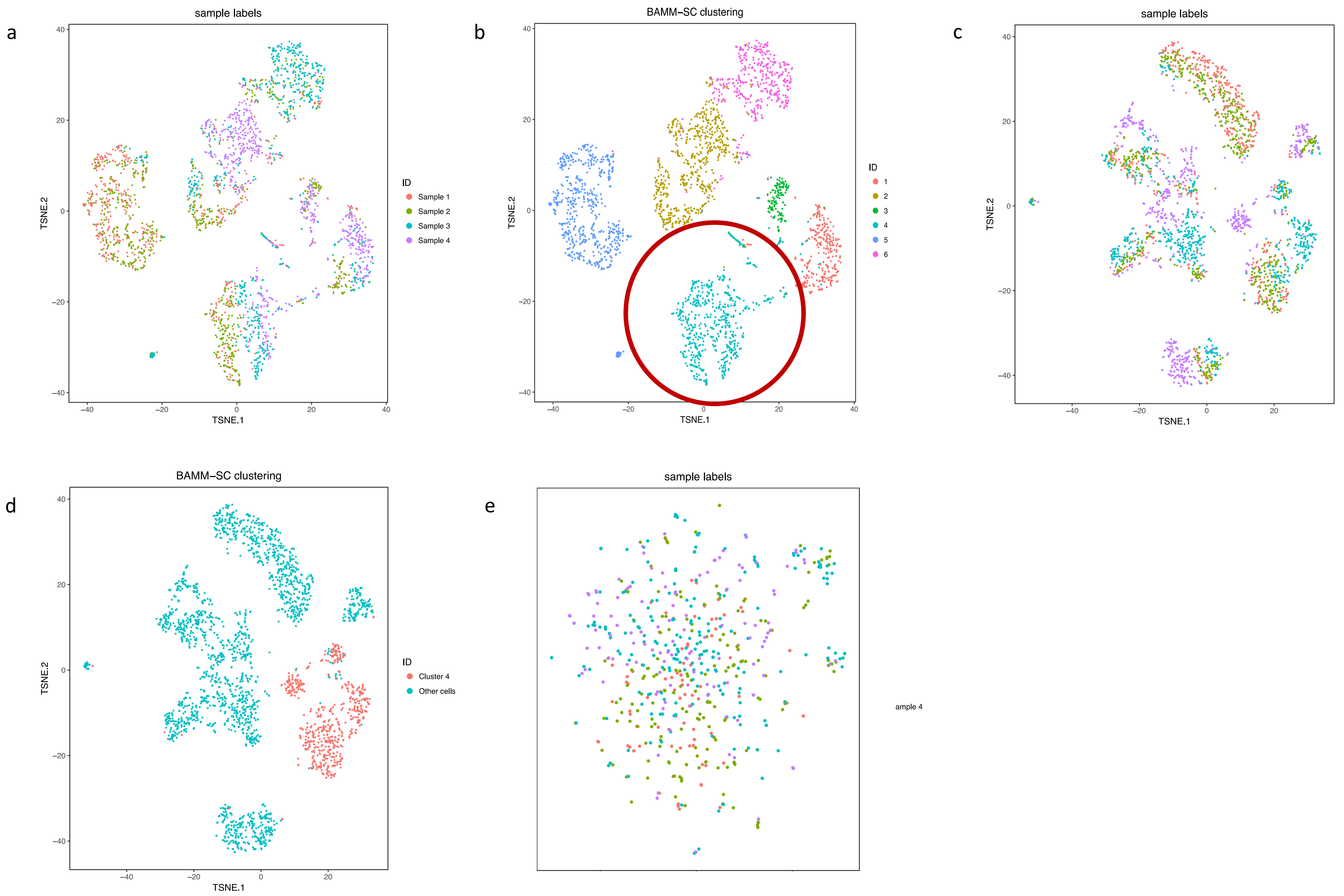

Supplementary Table 1. Sample information of three droplet-based scRNA-seq datasets

Dataset	Sample 1	Sample 2	Sample 3	Sample 4	Sample 5
Version of Cell Ranger					
Human PBMC	2.0.0	2.0.0	1.2.0	1.2.0	1.1.0
Mouse Lung	1.2.0	1.2.0	1.2.0	1.2.0	
Human Skin	1.3.0	1.3.0	2.0.0	2.0.0	2.0.0
Mean Reads per Cell					
Human PBMC	143,286	100,847	96,324	92,650	24,722
Mouse Lung	86,040	234,182	243,611	267,401	
Human Skin	215,718	168,951	157,677	51,669	107,424
Number of Cells					
Human PBMC	1,722	2,288	2,400	2,405	2,900
Mouse Lung	577	724	684	649	
Human Skin	960	1,713	686	2,240	1,636

Supplementary Table 2. Gene markers used to specify cell types in human PBMC samples.

Cell Types	Genes
CD8+ T cells	CD3+CD8A+CD4-
CD4+ T cells	CD3+CD8-CD4+IL2RA-IL7R+
B cells	CD3-CD19+MS4A1+
NK cells	NCAM1+NKG7+CD3-
CD14+ monocytes	CD3-CD19-CD14+HLA-
CD16+ monocytes	CD3-CD19-FCGR3A+
Dendritic cells	CD1C+CD14-HLA-FCER1A+

Supplementary Table 3. Gene markers used to specify cell types in mouse lung cell samples.

Cell Types	Genes
Macrophages	Ctss+Chil3+
Neutrophils	S100a8+S100a9+Il1b+
Endothelial	Lyve1+Egfl7+
Small airway Epithelial	Sftpc+Sftpd+Lyz1+
Club Cells	Scgb1a1+Scgb3a1+
Lymphocytes	Cd79b+Ms4a1+ / Gzma+Nkg7+

Supplementary Table 4. Gene markers used to specify cell types in human skin samples.

Cell Types	Genes
Smooth muscle cells	DES+
Suprabasal keratinocytes	KRT1+KRT10+
Basal keratinocytes	KRT14+KRT5+
Endothelial cells	VWF+
Fibroblasts	COL1A1+
Pericytes	RGS5+VWF-
Melanocytes	PMEL+
Mecrophages/Dendritic cells	AIF1+

Supplementary Table 5. Accuracy of the confusion matrix generated from different clustering methods for human PBMC, mouse lung and human skin samples. Clustering results are selected based on the highest ARI among 10 times analysis.

	Human PBMC	Mouse lung	Human skin
MNN + K-means	0.669	0.908	0.801
MNN + TSCAN	0.560	0.849	0.634
MNN + SC3	0.687	0.861	0.676
MNN + Seurat	0.511	0.598	0.775
CCA + K-means	0.673	0.947	0.797
CCA + TSCAN	0.452	0.827	0.638
CCA + SC3	0.528	0.811	0.591
CCA + Seurat	0.754	0.825	0.748
DIMM-SC	0.643	0.944	0.793
BAMM-SC	0.734	0.960	0.859

Supplementary Table 6. The correlation of estimated proportions of cells in each cell type between different clustering methods and flow cytometry in human PBMC sample 3. Clustering results are selected based on the highest ARI among 10 times analysis.

	Human PBMC
MNN + K-means	0.95
MNN + TSCAN	0.94
MNN + SC3	0.92
MNN + Seurat	0.76
CCA + K-means	0.97
CCA + TSCAN	0.60
CCA + SC3	0.88
CCA + Seurat	0.99
DIMM-SC	0.97
BAMM-SC	0.98

Supplementary Table 7. Performance of cluster stability measured by APN for human PBMC, mouse lung and human skin samples, respectively. We compared the clustering results based on full data (1,000 genes) to the clustering with randomly removing 100 genes. We repeated this step 10 times to calculate the APN. The APN values of BAMM-SC for each dataset are highlighted in bold font.

Method	Human PBMC	Mouse Lung	Human Skin
MNN + K-means	0.24	0.21	0.25
MNN + TSCAN	0.16	0.11	0.29
MNN + SC3	0.43	0.44	0.56
MNN + Seurat	0.14	0.20	0.24
CCA + K-means	0.29	0.16	0.28
CCA + TSCAN	0.60	0.37	0.67
CCA + SC3	0.69	0.23	0.64
CCA + Seurat	0.11	0.16	0.19
DIMM-SC	0.23	0.14	0.17
BAMM-SC	**0.23**	**0.07**	**0.16**

Supplementary Table 8. Performance of cluster tightness measured by silhouette width for human PBMC, mouse lung and human skin samples, respectively. The distance metric is Morisita dissimilarity. The silhouette width values of BAMM-SC for each dataset are highlighted in bold font.

	Human PBMC	Mouse Lung	Human Skin
MNN + K-means	0.40	0.33	0.16
MNN + TSCAN	0.18	0.34	0.16
MNN + SC3	0.14	0.32	0.11
MNN + Seurat	0.34	0.33	0.20
CCA + K-means	0.13	0.34	0.11
CCA + TSCAN	-0.03	0.23	0.03
CCA + SC3	-0.12	0.33	-0.02
CCA + Seurat	0.03	0.29	0.11
DIMM-SC	0.21	0.34	0.12
BAMM-SC	**0.35**	**0.35**	**0.17**

Supplementary Methods

Assumptions of BAMM-SC

First, cell type clusters are discrete, and each cell belongs to one cell type exclusively. We used a latent variable vector $\boldsymbol{Z_l}$ with element z_{jl} to represent the cell type label for the cell j in individual l. We assumed that the cell population consists of K distinct cell types. $z_{jl} \in \{1, 2, \dots, K\}$. Second, heterogeneity exists among different individuals and across different cell types. The heterogeneity of the same cell type among different individuals is smaller than the heterogeneity across different cell types within the same individual. This is reflected from the assumed mixture Dirichlet distribution, with a cell-type specific underlying log-normal distribution $LN(\mu_{ik}, \sigma_{ik}^2)$ for α_{ilk}, where σ_{ik}^2 quantifies the variability among different individuals for the same cell type k and different μ_{ik}'s (for different k's) specify the heterogeneity across different cell types. Thrid, cells of the same cell type share a similar gene expression pattern. "Similar gene expression pattern" assumption implies that cells from the same cell type shares a group of genes with more similar gene expression than those from different cell types. This "similar gene expression pattern" assumption is fundamental for all clustering methods. For a given individual l, if different single cells belong to the same cell type k, we assume their underlying distribution is the same $DIR(\alpha_{ilk} | \, i = 1, \dots, G)$.

Details of Gibbs sampler

Based on Bayes formula, we have the full posterior distribution as follows:

$$P(\boldsymbol{z}_{..}, \boldsymbol{\alpha}_{\dots} | \boldsymbol{x}_{\dots}) \propto P(\boldsymbol{x}_{\dots}, \boldsymbol{z}_{..} | \boldsymbol{\alpha}_{\dots}) \times \prod_{k=1}^{K} \prod_{i=1}^{G} Prior(\boldsymbol{\alpha_{i \cdot k}}) \times \prod_{k=1}^{K} Prior(\boldsymbol{\mu_{\cdot k}}) \times \prod_{k=1}^{K} Prior\left(\boldsymbol{\sigma_{\cdot k}^2}\right). \quad (1)$$

The complete log likelihood is:

$$\log P(\boldsymbol{z}_{..}, \boldsymbol{\alpha}_{\dots} | \boldsymbol{x}_{\dots}) = \sum_{l=1}^{L} \sum_{j=1}^{C_l} \sum_{k=1}^{K} I\left(z_{jl} = k\right) * \log\left\{\left(\prod_{i=1}^{G} \frac{\Gamma(x_{ijl} + \alpha_{ilk})}{\Gamma(\alpha_{ilk})}\right) \frac{\Gamma(|\boldsymbol{\alpha_{\cdot lk}}|)}{\Gamma(T_{jl} + |\boldsymbol{\alpha_{\cdot lk}}|)}\right\}$$
$$+ \sum_{k=1}^{K} \sum_{i=1}^{G} \sum_{l=1}^{L} \left\{-\log \alpha_{ilk} - \frac{(\log \alpha_{ilk} - \mu_{ik})^2}{2\sigma_{ik}^2}\right\} + \sum_{k=1}^{K} \sum_{i=1}^{G} \left\{-\frac{L}{2} \log \sigma_{ik}^2\right\}$$
$$+ NonInformativePrior(\mu_{..}) + \sum_{k=1}^{K} \sum_{i=1}^{G} logGammaPDF\left(\sigma_{ik}^2, a_k, b_k\right). \quad (2)$$

Here the hyper-prior parameters a_k and b_k can be pre-specified, or estimated from data via an empirical Bayes approach.

We will use Gibbs sample to iteratively update $\left\{z_{jl}\right\}_{1 \le j \le C_l, 1 \le l \le L}$, $\{\alpha_{ilk}\}_{1 \le i \le G, 1 \le l \le L, 1 \le k \le K}$. For a given pair of l and j, the conditional distribution for z_{jl} is a multinomial distribution, where

$$P\left(z_{jl} = k\right) = \frac{1}{Constant} * \left(\prod_{i=1}^{G} \frac{\Gamma\left(x_{ijl} + \alpha_{ilk}\right)}{\Gamma(\alpha_{ilk})}\right) \frac{\Gamma(|\boldsymbol{\alpha_{\cdot lk}}|)}{\Gamma\left(T_{jl} + |\boldsymbol{\alpha_{\cdot lk}}|\right)}. \quad (3)$$

Where the normalization constant is $\sum_{k=1}^{K} \left(\prod_{i=1}^{G} \frac{\Gamma(x_{ijl} + \alpha_{ilk})}{\Gamma(\alpha_{ilk})}\right) \frac{\Gamma(|\boldsymbol{\alpha_{\cdot lk}}|)}{\Gamma\left(T_{jl} + |\boldsymbol{\alpha_{\cdot lk}}|\right)}$.

We will use random walk Metropolis within Gibbs to iteratively update α_{ilk}. For a given triple of i, l and k, the conditional log likelihood for α_{ilk} is:

$$\log P(\alpha_{ilk}|\boldsymbol{x}_{\dots}, \boldsymbol{z}_{\cdot\cdot}) \propto \sum_{j=1}^{C_l} I(z_{jl} = k) * \log\left\{\left(\prod_{i=1}^{G} \frac{\Gamma(x_{ijl} + \alpha_{ilk})}{\Gamma(\alpha_{ilk})}\right) \frac{\Gamma(|\boldsymbol{\alpha}_{\cdot\boldsymbol{lk}}|)}{\Gamma(T_{jl} + |\boldsymbol{\alpha}_{\cdot\boldsymbol{lk}}|)}\right\} - \log \alpha_{ilk} - \frac{(\log \alpha_{ilk} - \mu_{ik})^2}{2\sigma_{ik}^2}. \quad (4)$$

Similarly, we will use random walk Metropolis within Gibbs to iteratively update σ_{ik}^2. For a given pair of i and k, the conditional log likelihood for σ_{ik}^2 is:

$$\log P(\sigma_{ik}^2| \dots) \propto \sum_{l=1}^{L} \left\{-\frac{(\log \alpha_{ilk} - \mu_{ik})^2}{2\sigma_{ik}^2}\right\} - \frac{L}{2}\log \sigma_{ik}^2 + logGammaPDF(\sigma_{ik}^2, a_k, b_k). \quad (5)$$

In random walk Metropolis algorithm, we adaptively select the step size of proposal distribution, to make sure that the acceptance rate is 20% ~ 30%.

Label switching issue

We can first run DIMM-SC on all single cells pooled from all individuals, to get initial values for z_{jl} and α_{ilk}. To get the initial values of σ_{ik}^2, we apply DIMM-SC to each individual separately and get α_{ik} for each individual. Then we can match α_{ik} from the same cell type across different individuals based on calculating Pearson correlation and L1 norm of the difference between α_{ik} from different individuals. Specifically, each cell cluster was assigned to the cluster from another individual, which has the highest correlation with its gene expression profile and the lowest L1 norm of the difference between α_{ik} from different individuals.

Data generation in simulation studies

We simulated drop-seq data with different heterogeneity among multiple individuals. In our posited hierarchical model, the log normal prior distribution $LN(\mu_{ik}, \sigma_{ik}^2)$ measures the heterogeneity of gene i in cell type k among multiple individuals and we also assume all σ_{ik}^2's follow a hyper-prior $Gamma(a_k, b_k)$. For simplicity, we used the mean of σ_{ik}^2 across all genes and all cell types to quantify the individual level heterogeneity.

In our simulation set-up, the UMI count matrix was sampled from a proposed Dirichlet mixture model. Specially, for a fixed total number of cell clusters K, we first pre-defined the values of μ_{ik}, a_k and b_k for each cell cluster, and then sampled the σ_{ik}^2 from the Gamma distribution $Gamma(a_k, b_k)$. The matrix of σ_{ik}^2 is fixed in each simulation iteration. Next, we sampled $\alpha_{i.k}$ from a Log Normal distribution $LN(\mu_{ik}, \sigma_{ik}^2)$ for each gene and each cell cluster. Proportion $\boldsymbol{p}_{\cdot\boldsymbol{jl}} = (p_{1jl}, p_{2jl}, \dots, p_{Gjl})$ was sampled from a Dirichlet distribution with parameter vector $\boldsymbol{\alpha}_{\cdot\boldsymbol{l(k)}} = (\alpha_{1lk}, \alpha_{2lk}, \dots, \alpha_{Glk})$. Last, we sampled the UMI count vector $\boldsymbol{x}_{\cdot\boldsymbol{jl}} = (x_{1jl}, x_{2jl}, \dots, x_{Gjl})$ for the cell j and individual l from the multinomial distribution $Multi(T_{jl}, \boldsymbol{p}_{\cdot\boldsymbol{jl}})$. We fixed T_{jl} as a constant across all cells and individuals.

In our simulation analysis, we simulated 100 datasets and reported the corresponding ARIs. All clustering methods were run under default parameters. Specifically, when using Seurat to correct batch effect, we randomly chose 5 simulated datasets among 100, used R function "DimHeatmap" and "MetageneBicorPlot" to plot the correlation strength of each canonical correlation (CC). Based on these plots, we decided which CCs to use and align, and fixed it for all 100 simulated datasets.
