# Supplementary material for: A Bayesian mixture model for clustering droplet-based single-cell transcriptomic data from population studies
Source: Nat Commun. 2019 Apr 9;10:1649. doi: 10.1038/s41467-019-09639-3 (PMC6456731; doi:10.1038/s41467-019-09639-3)
Supplement: Supplementary file 2 — Reporting Summary [file 41467_2019_9639_MOESM2_ESM.pdf]

nature research

Corresponding author(s): DBPR NCOMMS-18-24085C

Last updated by author(s): Mar 1, 2019

Reporting Summary

Nature Research wishes to improve the reproducibility of the work that we publish. This form provides structure for consistency and transparency in reporting. For further information on Nature Research policies, see Authors & Referees and the Editorial Policy Checklist.

Statistics

For all statistical analyses, confirm that the following items are present in the figure legend, table legend, main text, or Methods section.

n/a	Confirmed	
☐	☒	The exact sample size (*n*) for each experimental group/condition, given as a discrete number and unit of measurement
☐	☒	A statement on whether measurements were taken from distinct samples or whether the same sample was measured repeatedly
☒	☐	The statistical test(s) used AND whether they are one- or two-sided<br>*Only common tests should be described solely by name; describe more complex techniques in the Methods section.*
☒	☐	A description of all covariates tested
☐	☒	A description of any assumptions or corrections, such as tests of normality and adjustment for multiple comparisons
☐	☒	A full description of the statistical parameters including central tendency (e.g. means) or other basic estimates (e.g. regression coefficient) AND variation (e.g. standard deviation) or associated estimates of uncertainty (e.g. confidence intervals)
☒	☐	For null hypothesis testing, the test statistic (e.g. *F*, *t*, *r*) with confidence intervals, effect sizes, degrees of freedom and *P* value noted<br>*Give P values as exact values whenever suitable.*
☐	☒	For Bayesian analysis, information on the choice of priors and Markov chain Monte Carlo settings
☐	☒	For hierarchical and complex designs, identification of the appropriate level for tests and full reporting of outcomes
☐	☒	Estimates of effect sizes (e.g. Cohen's *d*, Pearson's *r*), indicating how they were calculated

Our web collection on statistics for biologists contains articles on many of the points above.

Software and code

Policy information about availability of computer code

Data collection	We used the standard Cell Ranger's pipelines from 10X Genomics to analyze sequencing data generated from Chromium Single Cell 3' RNA-seq libraries to get the UMI count matrix.
Data analysis	BAMM-SC, including all source and example code, is freely available as an R package with a detailed tutorial at http://github.com/CHPGenetics/BAMMSC.

For manuscripts utilizing custom algorithms or software that are central to the research but not yet described in published literature, software must be made available to editors/reviewers. We strongly encourage code deposition in a community repository (e.g. GitHub). See the Nature Research guidelines for submitting code & software for further information.

Data

Policy information about availability of data

All manuscripts must include a data availability statement. This statement should provide the following information, where applicable:

- Accession codes, unique identifiers, or web links for publicly available datasets
- A list of figures that have associated raw data
- A description of any restrictions on data availability

This study uses publicly available scRNA-seq datasets. Both human PBMC (sample 5) and purified CD19+ B cell scRNA-seq data that support the findings of this study are available at http://support.10xgenomics.com/single-cell-gene-expression/datasets. The raw and preprocessed experimental test datasets (human PBMCs, mouse lung and human skin tissues) are publicly available at http://github.com/CHPGenetics/BAMMSC. All other relevant data are available upon request.

Field-specific reporting

Please select the one below that is the best fit for your research. If you are not sure, read the appropriate sections before making your selection.

☒ Life sciences ☐ Behavioural & social sciences ☐ Ecological, evolutionary & environmental sciences

For a reference copy of the document with all sections, see nature.com/documents/nr-reporting-summary-flat.pdf

Life sciences study design

All studies must disclose on these points even when the disclosure is negative.

Sample size	No statistical methods were used to predetermine sample size. Large numbers of cells in each individual constitute a sufficient sample size for accurate estimation.
Data exclusions	No data were excluded from the analyses.
Replication	In this study, we applied our method on each real dataset and repeated it 10 times to evaluate the stability of performance. The raw and preprocessed test datasets and related R codes are publicly available at http://github.com/CHPGenetics/BAMMSC.
Randomization	Randomization is not relevant to our study. Methods involved in this study focus on analyzing data from only one experimental group/ condition.
Blinding	Blinding is not relevant to our study. Methods involved in this study focus on analyzing data from one experimental group/condition.

Reporting for specific materials, systems and methods

We require information from authors about some types of materials, experimental systems and methods used in many studies. Here, indicate whether each material, system or method listed is relevant to your study. If you are not sure if a list item applies to your research, read the appropriate section before selecting a response.

Materials & experimental systems

n/a	Involved in the study	
☒	☐	Antibodies
☒	☐	Eukaryotic cell lines
☒	☐	Palaeontology
☐	☒	Animals and other organisms
☐	☒	Human research participants
☒	☐	Clinical data

Methods

n/a	Involved in the study	
☒	☐	ChIP-seq
☒	☐	Flow cytometry
☒	☐	MRI-based neuroimaging

Animals and other organisms

Policy information about studies involving animals; ARRIVE guidelines recommended for reporting animal research

Laboratory animals	We collected lung mononuclear cells from 2 naive mice and 2 streptococcus pneumonia infected mice. Lung single cell suspension from naive and infected mice were subjected to scRNA-seq library preparation protocol.
Wild animals	This study did not involve wild animals.
Field-collected samples	This study did not involve samples collected from the field.
Ethics oversight	University of Pittsburgh Institutional Animal Care and Use Committee

Note that full information on the approval of the study protocol must also be provided in the manuscript.

Human research participants

Policy information about studies involving human research participants

Population characteristics	9 healthy control participants were included in our study.
Recruitment	After informed consent, samples were obtained from healthy participants. There is no self-selection bias, and how these participants recruited will not impact the results of our study.

Ethics oversight	University of Pittsburgh Institutional Review Board

Note that full information on the approval of the study protocol must also be provided in the manuscript.
